# Health effects of micronutrient fortified dairy products and cereal food for children and adolescents: A systematic review

**DOI:** 10.1371/journal.pone.0210899

**Published:** 2019-01-23

**Authors:** Klaus Eichler, Sascha Hess, Claudia Twerenbold, Magalie Sabatier, Flurina Meier, Simon Wieser

**Affiliations:** 1 Winterthur Institute of Health Economics, Zurich University of Applied Sciences, Winterthur, Switzerland; 2 Nestlé Research Center, Public Health Department, Lausanne, Switzerland; University of Ghana, GHANA

## Abstract

**Introduction:**

Micronutrient (MN) deficiencies cause a considerable burden of disease for children in many countries. Dairy products or cereals are an important food component during adolescence. Fortification of dairy products or cereals with MN may be an effective strategy to overcome MN deficiencies, but their specific impact on health in this age group is poorly documented.

**Methods:**

We performed a systematic review and meta-analysis (registration number CRD42016039554) to assess the impact of MN fortified dairy products and cereal food on the health of children and adolescents (aged 5–15 years) compared with non-fortified food. We reviewed randomised controlled trials (RCT) using electronic databases (MEDLINE, EMBASE, Cochrane library; latest search: January 2018), reference list screening and citation searches. Three pairs of reviewers assessed 2048 studies for eligibility and extracted data. We assessed the risk of bias and applied GRADE to rate quality of evidence.

**Results:**

We included 24 RCT (often multi MN fortification) with 30 pair-wise comparisons mainly from low- and middle income countries. A very small and non-significant increase of haemoglobin values emerged (0.09 g/dl [95%-CI: -0.01 to 0.18]; 13 RCT with iron fortification; very low quality of evidence). No significant difference was found on anaemia risk (risk ratio 0.87 [95%-CI: 0.76 to 1.01]; 12 RCT; very low quality), but a significant difference in iron deficiency anaemia favouring fortified food was found (risk ratio 0.38 [95%-CI: 0.18 to 0.81]; 5 RCT; very low quality). Similar effects were seen for fortified dairy products and cereals and different fortification strategies (mono- vs. dual- vs. multi-MN). Follow-up periods were often short and the impact on anthropometric measures was weak (low quality of evidence) Very low quality of evidence emerged for the improvement of cognitive performance, functional measures and morbidity.

**Conclusions:**

Fortification of dairy products and cereal food had only marginal health effects in our sample population from 5–15 years. Further evidence is needed to better understand the health impact of fortified dairy products and cereals in this age group.

**Systematic review registration:**

The study protocol was registered with the International Prospective Register of Systematic Reviews (PROSPERO) on 26 May 2016 (registration number CRD42016039554).

## Introduction

Micronutrient (MN) deficiencies cause a considerable burden of disease for children in many countries with impaired physical and cognitive development, as well as increased morbidity and mortality [1].

Different strategies have been shown to be effective in reducing MN deficiencies for different target groups and are proposed in recommendations and guidelines [2–4]. One approach is to improve daily diet by increasing meat and milk intake to improve consumption of some MNs, for example iron and calcium. However, this is often not possible due to limited availability, affordability or cultural and religious reasons [5]. Another option is MN supplementation (e.g. vitamin A capsules administered at defined intervals). Fortification is also widely applied to reduce MN deficiencies in general populations of middle and low income countries, as well as in high income countries [2]. Food fortification is the addition of one or more essential nutrients to a food, whether or not it is normally contained in the food, for the purpose of preventing or correcting a demonstrated deficiency of one or more nutrients in the population or specific population groups [6]. This also includes fortification of centrally processed staple foods or other packaged food products. Fortification can also refer to the so-called home fortification where a mixture of MNs is added to any food by simply sprinkling the MN powder over the food before consumption. Fortification strategies include single, dual or multi MN fortification. Examples of carriers for fortification are salt, sugar, flour, milk, oil or condiments.

From a public health perspective, an optimal physical and mental development in the age group 5–15 years is a prerequisite to benefiting completely from education at school [7]. Furthermore, it is also important for adolescents starting employment and for young women who enter child bearing age to confer to their future offspring an optimal nutritional status. Energy adequate nutrition, rich in MN, can contribute to the improved health status of this population group in developing countries provided other appropriate public health measures, such as improved sanitation and a safe water supply are simultaneously undertaken. Ultimately, this may be a feasible way of escaping the malnutrition trap [8].

Diet of schoolchildren and adolescents in developing countries is limited in diversity. In a recent review of 31studies [9] cereal-based foods (e.g. porridge, bread, biscuits) were reported as one of their main important sources of energy beside other plant-based diets (e.g. roots and tubers) with limited animal source foods. Inversely, depending on the locality, the consumption of milk and dairy products was highly variable even though they are an essential source of calcium for bone health. Nevertheless, dairy products and cereals are often used in school feeding programmes of low and middle income countries and might have an important health impact if they are fortified [10].

Several systematic reviews have assessed the impact of fortification for different target populations and different nutrient carriers [2, 11–15]. For example, research into the fortification of milk and cereals for infants and children from 6–59 months, has shown that multi-micronutrient (MMN) fortification can reduce anaemia rates [14, 15]. However, some questions remain unanswered: the specific impact of fortified dairy products and cereals for children and adolescents from 5 up to 15 years is less well documented than it is for infants and toddlers. In addition, MMN fortification seems to be more effective than single MN fortification [11, 16], but this may not always be the case [17] and the specific impact of MMN fortification on children and adolescents from 5 up to 15 years consuming fortified dairy products and cereals is unclear.

Thus, we performed a systematic review and meta-analysis of randomised controlled trials to assess the impact of MN fortified dairy products and cereal food on the health of children and adolescents (aged 5–15 years) compared with non-fortified food.

## Methods

Our systematic review took into account critical methodological issues of current guidelines for performing [18, 19] and reporting of systematic reviews (PRISMA checklist: S1 Table) [20, 21]. The review protocol was registered with the International Prospective Register of Systematic Reviews (PROSPERO) on 26 May 2016 (registration number CRD42016039554; http://www.crd.york.ac.uk/prospero/; study protocol: S1 SP).

### Inclusion and exclusion criteria

In keeping with our research question we defined the following inclusion criteria (for further details see S2 Table):

#### Population

Children (aged 5 to 12 years) and adolescents (aged 12 to 15 years) of both sexes and from all risk groups. The upper age limit was set at 15 years, as it matches the (de jure) school leaving age and the start of employment in most low- to middle-income countries in Asia and Africa [22]. Studies with mixed population groups are included only if the majority of participants are within the age range of 5 to 15 years.

#### Intervention

Centrally-processed fortified dairy products and fortified cereals, using any fortification strategy. Dairy products included fortified fresh milk; centrally processed milk or other dairy products (such as yoghurts, milk powder and cheese). Cereals included, for example, fortified wheat flour or maize (corn). Examples of food preparations with fortified cereals are porridge, bread or biscuits. We included any MN for fortification (for example iron, vitamins, zinc, iodine, calcium, folate) or their combination.

#### Control intervention

Non-fortified food; co-interventions (e.g. deworming) are accepted, if they were applied in the intervention and the control group.

#### Outcome

Blood parameters with direct health impact were the primary outcomes as per protocol: haemoglobin values (g/dl; conversion to g/L with factor 10) and anaemia rates. Secondary outcomes included iron stores (ferritin), Z-scores (those provided by the authors) as growth indicators (for wasting WHZ: weight-for-height-Z, for stunting HAZ: height-for-age-Z), other direct health outcome (body weight; functional status; cognitive development; school performance; quality of life; morbidity; mortality); acceptability of fortified products and harms of fortified foods.

#### Study designs

Randomised controlled trials (RCTs) of any follow-up time.

We excluded nutritional interventions based solely on supplementation, other fortified staple foods (e.g. fortified salt), home fortification, pulses, soy-based products (tofu; soya milk) and interventions with fortified rice, as fortification of rice with several MN is being addressed by an ongoing Cochrane review [23]. We did not include surrogate parameters (for example zinc, iodine or vitamin blood levels) as an outcome. Surrogate parameters can be relevant from a nutritional science perspective, but they do not allow comparing the direct health impact of the intervention by themselves.

### Search strategy

With the support of a medical information specialist, we systematically searched for studies using electronic databases: MEDLINE (OVID Interface; search strategy Table 1), Embase and the COCHRANE-Library (from inception to January 2018; no language restriction). We also screened the homepages of organisations engaged in nutrition projects in low- and middle-income countries (e.g. WHO; United Nations [World Food Programme, UNICEF, Millennium Development Goals]; The World Bank; Nutrition International; Bill & Melinda Gates Foundation). Furthermore, we searched Google Scholar and conducted reference screening (for included RCT and for relevant systematic reviews) and citation searches.

**Table 1:** Medline electronic search strategy.

No.	Searches	Results
1	((exp milk/ or exp cheese/ or Edible Grain/) and fortif*.ti,ab.) or (fortif* adj3 (milk or yoghurt or yogurt or yoghourt or cheese* or cereal* or gruel or porridge or muesli or musli or flour or bread or biscuit* or "sweet rolls" or rusk or maize or corn or wheat* or oat* or millet or sorghum or rye or buckwheat)).ti,ab.	2626
2	adolescent/ or child/ or (child* or adolescent*).ti,ab.	2836894
3	1 and 2	672

Database: Ovid MEDLINE In-Process & Other Non-Indexed Citations, Ovid MEDLINE Daily and Ovid MEDLINE 1946 to Present; ti,ab: Title/Abstract; http://ovidsp.tx.ovid.com/sp-3.20.0b/ovidweb.cgi[25.05.2016 12:47:18].

### Study selection and data extraction

Prior training sessions took place to ensure high consistency between reviewers (chance-adjusted kappa statistics: 0.95). Three reviewer pairs were assigned to one third of the retrieved references, each, and screened titles and abstracts for relevance. Within each pair, screening was done independently by each reviewer. Full text copies were assessed for a final decision by one reviewer, with decisions confirmed by a second reviewer. Disagreements in both steps were resolved by consensus. Unclear cases were discussed with a senior reviewer. If data from a specific population were published in several papers or if follow-up data were presented, each population was included only once.

Data were extracted by one reviewer in an Excel database and confirmed by a second reviewer. Again, disagreements were resolved by consensus. Unclear cases were discussed with a senior reviewer. We extracted data on study and participant details; intervention and control (e.g. daily amount of fortified MN, determined as the daily difference between the intervention and control group); and study results (details on extracted data in the study protocol: http://www.crd.york.ac.uk/prospero/). For six studies with more than two intervention groups and one control group [24–29] we combined the intervention groups to create a single pairwise comparison (Cochrane Handbook; Chapter 16.5.4 [18]).

One reviewer assessed the risk of bias in individual studies using criteria derived from the Cochrane risk of bias tool (Cochrane Handbook, Chapter 8 [18]: generation of random sequence and concealment of allocation [selection bias]; blinding of participants and personnel [performance bias]; blinding of outcome assessment [detection bias]; incomplete outcome data [attrition bias]; and selective reporting [reporting bias]; updated risk of bias criteria see S5 Table). To make an overall rating of confidence in estimates of effects, one reviewer applied the GRADE approach and rated the quality of evidence of specific effects [30]. Risk of bias assessment and GRADE rating were confirmed by a second reviewer and disagreements were resolved by consensus.

### Statistical analysis

For the pooling of continuous variables, we calculated weighted mean differences (WMD) and 95%-confidence intervals (CI) using the inverse variance method. For example, when analysing the primary outcome haemoglobin change we used the mean change in the intervention and the control group and their pooled standard deviation (SD). For the pooling of binary data, we calculated risk ratios and 95%-CI. For cluster RCT, we adjusted for intra-cluster correlation, where authors had not reported adjustment (Cochrane Handbook, Chapter 16.3 [18, 31]). For the meta-analysis of haematological outcomes, we included only studies that explored iron fortification. One study explored the effect of vitamin A without iron and reported haematological outcomes. To isolate a potential effect of vitamin A on haematological outcomes, we reported the results of this study separately.

Heterogeneity between trials was calculated with I^2^, that is the percentage of the total variation in estimated effects due to heterogeneity rather than chance [32] (0%-40% might not be important; 30%-60% may represent moderate heterogeneity; 50%-90% may represent substantial heterogeneity; 75%-100%: considerable heterogeneity). Since we found at least moderate statistical heterogeneity between trials, we applied a random effects model [33].

When the sample size decreased during the study, we used the lower sample size at the end of the study. Using as a denominator the total number of participants who had data recorded for the particular outcome, we avoided ending up with an apparently high precision (Cochrane Handbook, Chapter 16.2 [18]). If mean haemoglobin change per group and SD was not reported, we calculated change as the difference between baseline and final values for intervention and control group. We imputed the change-from-baseline SD using a correlation coefficient (Cochrane Handbook; chapter 16.1.3.2 [18]). If only 95%-CI of mean values were reported, we converted them to SD assuming normal distribution [34]. To test the results for robustness, we also calculated WMD for the final haemoglobin values of both randomised study arms at the end of follow-up. Where authors reported only medians for continuous data, we did not include these data in a meta-analysis, but reported the distribution of median values and inter-quartile-ranges (IQR).

To assess secondary research questions as defined in the protocol and to explore the influence of possible modifying factors on the outcome, we divided our dataset into pre-specified subgroups (fortified dairy products vs. fortified cereal food; single- vs. dual/multi-micronutrient fortification strategy; studies from high vs. studies from low/middle-income countries; studies with low risk of bias vs. studies with intermediate/high risk of bias). Finally, we performed a meta-regression analysis weighted for the inverse of the variance of the outcome to explain heterogeneity further (Cochrane Handbook; Chapter 9.6.4 [18]. Using this approach, we evaluated the unique contribution of other a priori chosen independent factors (mean haemoglobin level at the start of the study; daily amount of consumed MN from fortified food; length of study follow-up; completeness of study follow-up) on the primary outcome (dependent variable).

For parametric and non-parametric tests p-values <0.05 were considered significant. Analyses were performed using the STATA SE 14.2 software package (StataCorp. 2015. Stata Statistical Software, College Station, Texas, USA).

## Results

### Description of included studies and populations

**Fig 1:**
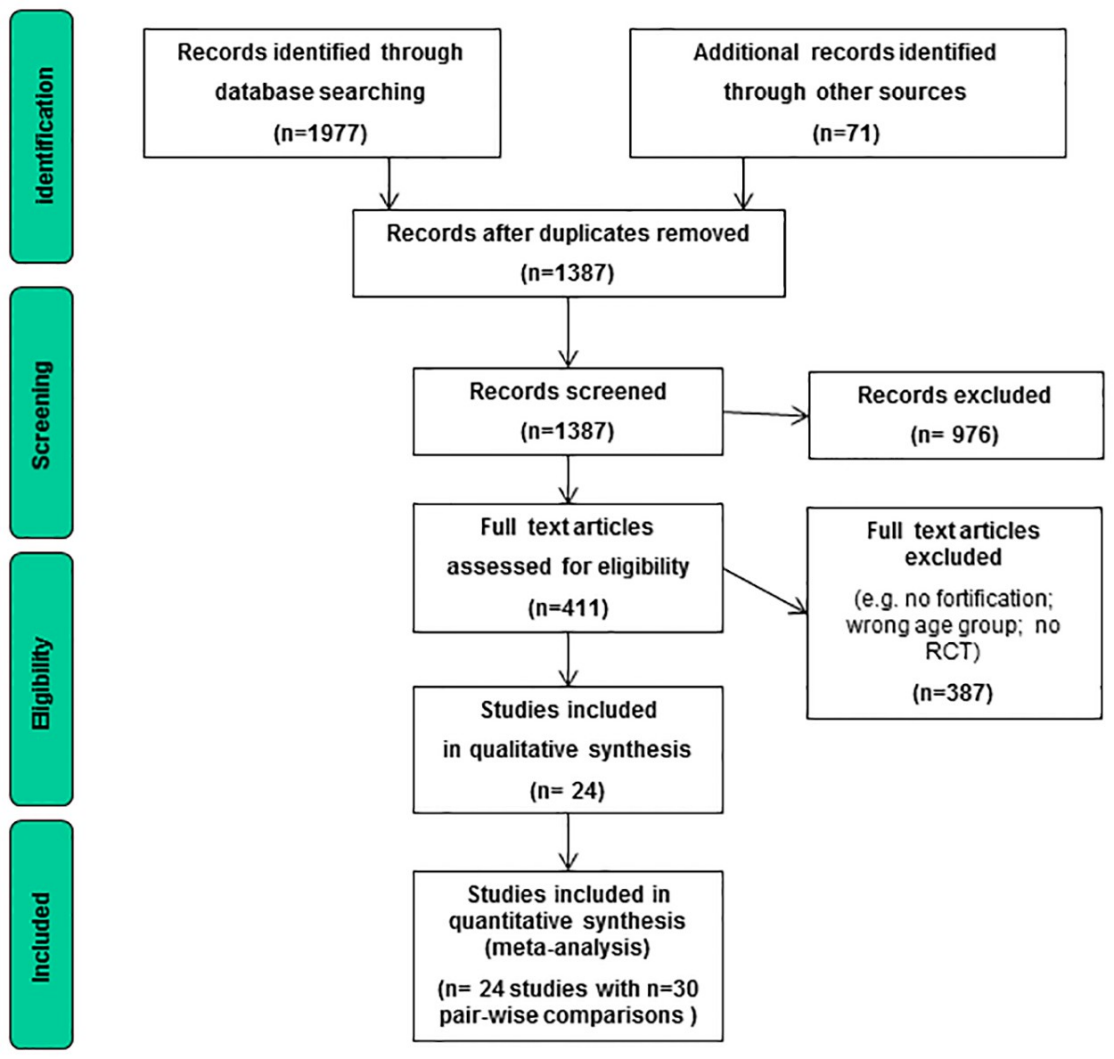
PRISMA flow diagram of the systematic review.

Our searches retrieved 2048 potentially relevant studies (PRISMA flow diagram: Fig 1). Twenty-four RCTs (n = 11 fortified dairy products [27, 35–44]; n = 13 fortified cereals [24–26, 28, 29, 31, 45–51]) fulfilled the inclusion criteria, provided suitable data and were included in our main analysis (studies included: Table 2; examples of excluded studies with reasons for exclusion: S3 Table). Seven of the 24 trials were cluster-randomised trials [31, 35–38, 42, 44]. The 24 RCTs reported about 9,367 children and adolescents, mostly from low- and middle-income countries (17 studies from Asia [27, 28, 31, 35–42, 44, 46, 48–51], six from Africa [24–26, 29, 45, 47]; and one from Australia, which provided only height and weight change data [43]. 15 of 24 RCTs were industry funded [24–27, 29, 35–37, 39, 41, 43, 45, 47, 49, 50], six of which in combination with public funding [24, 37, 39, 47, 49, 50]. Study population sizes varied from n = 88 to n = 1010 participants (mean: n = 390). Follow up periods were generally short (mean follow up: 8 months; range: 12 weeks—2 years), but the completeness of follow-up was generally high (median 94%; IQR: 89%-95%).

**Table 2:** Details of included 30 pair-wise comparisons of 24 primary studies.

Study	Population	Intervention food	Control food	Outcome measures	Comment
Author, year: Andang’o, 2007 [24] Design: RCT Follow-up (years): 0.42 Sponsor: public & industry funding	Country: Kenya Setting: school feeding intervention Target population: nursery and first year of school children Age (mean):6 years (range: 3 to 8) Males (%): 50	*n = 387* iron-fortified whole maize porridge; multi MN strategy MN applied: iron, VitA, VitB1, VitB2, VitB3	*n = 128* non-fortified whole maize porridge	Hb, anemia, ferritin, iron deficiency anaemia	4 arm trial Sponsors: Unilever Food and Research Institute; Akzo Nobel Chemicals; Netherlands Organisation for Scientific Research NWO/WOTRO
Author, year: Bardosono, 2009 [35] Design: cluster RCT Follow-up (years): 0.42 Sponsor: no info	Country: Indonesia Setting: school feeding intervention Target population: school children from low socioeconomic urban areas Age (mean):7.9 years (range: 7 to 9) Males (%): 52	*n = 61* iron-zinc-fortified milk; dual MN strategy MN applied: iron, zink	*n = 62* non-fortified milk	Hb, anemia, ferritin, anthropometrics (height change; weight change; WAZ-score; HAZ-score), function (cognitive; physical)	Sponsors: no info
Author, year: Bass, 2007 [43] Design: RCT Follow-up (years): 0.42 Sponsor: industry funding	Country: Australia Setting: school feeding intervention Target population: school children from Melbourne Age (mean):9 years (range: 7 to 11) Males (%): 100	*n = 21* calcium fortified muffins or cookies (using milk minerals); single MN strategy MN applied: calcium	*n = 26* non-fortified muffins or cookies	anthropometrics (height change; weight change)	4 arm trial: data are extracted for the two non-exercise groups Sponsors: Dairy Research and Development Corporation; Murray Goulbourn Co-op Pty.
Author, year: Bass, 2007 [43] Design: RCT Follow-up (years): 0.42 Sponsor: industry funding	Country: Australia Setting: school feeding intervention Target population: school children from Melbourne Age (mean):9 years (range: 7 to 11) Males (%): 100	*n = 20* calcium fortified muffins or cookies (using milk minerals) plus exercise; single MN strategy MN applied: calcium	*n = 21* non-fortified muffins or cookies plus exercise	anthropometrics (height change; weight change)	4 arm trial: data are extracted for the two groups applying moderate impact exercise Sponsors: Dairy Research and Development Corporation; Murray Goulbourn Co-op Pty.
Author, year: Bouhouch, 2016 [29] Design: RCT Follow-up (years): 0.42 Sponsor: partly industry funding	Country: Marocco Setting: school feeding intervention Target population: third- through sixth-grade aged children school children Age (mean):7.3 years (range: no info) Males (%): 49	*n = 226* iron fortified biscuits; single MN strategy MN applied: iron	*n = 117* non-fortified biscuits	Hb, anemia, ferritin, iron deficiency anaemia, cognition	4 arm trial Sponsors: Swiss National Science Foundation; Dr. Paul Lohmann GmbH; Hug AG
Author, year: Camargo, 2012 [42] Design: cluster RCT Follow-up (years): 0.42 Sponsor: public funding	Country: Mongolia Setting: school feeding intervention Target population: 3rd and 4th grade schoolclasses Age (mean):9.9 years (range: 9 to 11) Males (%): 52	*n = 141* Vit. D fortified milk; single MN strategy MN applied: VitD	*n = 103* non-fortified milk	morbidity, adverse events, anthropometrics (height change, weight change, BMI)	The Blue Sky Study (additional data extracted from other publications [52, 53] Sponsors: anonymous foundation; Massachusetts General Hospital Center, Boston, Massachusetts
Author, year: Du, 2004 [37] Design: cluster RCT Follow-up (years): 0.42 Sponsor: public & industry funding	Country: China Setting: school feeding intervention Target population: urban school girls Age (mean):10 years (range: 10 to 10) Males (%): 0	*n = 120* calcium and VitD fortified milk; dual MN strategy MN applied: calcium, VitD	*n = 117* no intervention (i.e. no unfortified milk)	anthropometrics (height change; weight change)	3 arm trial Sponsors: Australian Dairy Research and Development Corporation; Nestle Foundation
Author, year: Hieu, 2012 [50] Design: RCT Follow-up (years): 0.42 Sponsor: public funding	Country: Vietnam Setting: school feeding intervention Target population: no info Age (mean):7.7 years (range: 6 to 9) Males (%): 46	*n = 100* MMN fortified biscuits; multi MN strategy MN applied: iron, zink, iodine, VitA, VitB6, VitB12, VitC, VitD, VitK, calcium (plus other MN)	*n = 95* non-fortified biscuits	Hb, anemia, ferritin, iron deficiency anaemia	3 arm trial with one supplementation group (Fe-tablet; data not extracted) Sponsors: Decentralized French Cooperation; Sight and Life; Institute of Research and Development, France
Author, year: Kuriyan, 2016 [41] Design: RCT Follow-up (years): 0.42 Sponsor: industry funding	Country: India Setting: school feeding intervention Target population: children from rural school, low to middle socio-economic bracket Age (mean):8.2years (range: 7 to 10) Males (%): 23	*n = 111* MMN fortified milk; multi MN strategy MN applied: iron, zink, iodine, VitA, VitB6, VitB12, VitC, VitD, calcium (plus other MN)	*n = 114* non-fortified milk	Hb, anemia, ferritin, iron deficiency anaemia, anthropometrics (height change; weight change), function (cognitive; physical), morbidity, adverse events	Sponsors: Mondelez International, Inc. (formerly Kraft Foods Global, Inc.)
Author, year: Le, 2007 [51] Design: RCT Follow-up (years): 0.42 Sponsor: public funding	Country: Vietnam Setting: school feeding intervention Target population: children from rural schools Age (mean):7.3 years (range: 6 to 8) Males (%): 50	*n = 86* iron fortified noodles; single MN strategy MN applied: iron	*n = 82* non-fortified noodles	Hb, anemia, ferritin, anthropometrics, morbidity	5 arm intervention (data for iron supplemenation not extracted) Sponsors: Neys-van Hoogstraten Foundation; Ellison Medical Foundation and the Ministry of Education and Training, Vietnam
Author, year: Le, 2007 [51] Design: RCT Follow-up (years): 0.42 Sponsor: public funding	Country: Vietnam Setting: school feeding intervention Target population: children from rural schools Age (mean):7.3 years (range: 6 to 8) Males (%): 49	*n = 79* iron fortified noodles with mebendazole; single MN strategy MN applied: iron	*n = 79* non-fortified noodles with mebendazole	Hb, anemia, ferritin, anthropometrics, morbidity	5 arm intervention (data extracted for (iron&deworming) vs deworming; data for iron supplemenation not extracted) Sponsors: Neys-van Hoogstraten Foundation; Ellison Medical Foundation and the Ministry of Education and Training, Vietnam
Author, year: Muthayya, 2012 [49] Design: RCT Follow-up (years): 0.42 Sponsor: public & industry funding	Country: India Setting: school feeding intervention Target population: children from 1 urban primary school, 2 rural primary schools Age (mean):10.75 years (range: 6 to 15) Males (%): 53	*n = 186* iron fortified chapathi; single MN strategy MN applied: iron	*n = 193* non-fortified chapathi	Hb, anemia, ferritin, iron deficiency anaemia, anthropometrics (height change; weight change), function (cognitive; physical)	Sponsors: Department of Biotechnology, Ministry of Science and Technology, Government of India; AkzoNobel; St. John s National Academy of Health Sciences, Bangalore, India.
Author, year: Nestel, 2004 [28] Design: RCT Follow-up (years): 0.42 Sponsor: public funding	Country: Sri Lanka Setting: community household setting Target population: primary schoolers Age (mean):8.6 years (range: 6 to 11) Males (%): 54	*n = 359* iron fortified (electrolytic) flour; single MN strategy MN applied: iron	*n = 191* non-fortified flour	Hb, anemia	3 arm trial Sponsors: USAID; OMNI and MGL project
Author, year: Neyestani, 2013 [38] Design: cluster RCT Follow-up (years): 0.42 Sponsor: public funding	Country: Iran Setting: school feeding intervention Target population: children from primary schools Age (mean):no info (range: 10 to 12) Males (%): 50	*n = 80* Calcium-VitD-fortified milk; dual MN strategy MN applied: calcium, VitD	*n = 53* non-fortified plain milk	anthropometrics (weigth change)	6 arm trial (only data for 2 arms suitable (fortified vs. plain milk) and extracted Sponsors: National Nutrition and Food Technology Research Institute (NNFTRI) of Iran
Author, year: Nga, 2011 [48] Design: RCT Follow-up (years): 0.42 Sponsor: public funding	Country: Vietnam Setting: school feeding intervention Target population: children from rural schools Age (mean):7.6 years (range: 6 to 8) Males (%): 48	*n = 114* MMN fortified biscuits; multi MN strategy MN applied: iron, zink, iodine, VitA, Thiamin, Riboflavin, VitB6, VitB12, VitC, folic acid, VitD, calcium (plus other MN)	*n = 118* non-fortified biscuits	Hb, anemia, ferritin, anthropometrics (HAZ-score; WAZ-score; WHZ-score), function (cognitive; physical), morbidity	2x2 factorial design with 4 groups (total N = 510); here data extracted for fortified biscuits vs placebo (n = 256); some data from additional publication [54] Sponsors: Neys-van Hoogstraten Foundation, The Netherlands; Ellison Medical Foundation
Author, year: Nga, 2011 [48] Design: RCT Follow-up (years): 0.42 Sponsor: public funding	Country: Vietnam Setting: school feeding intervention Target population: children from rural schools Age (mean):7.6 years (range: 6 to 8) Males (%): 48	*n = 118* MMN fortified biscuits + deworming (Albendazole); multi MN strategy MN applied: iron, zink, iodine, VitA, Thiamin, Riboflavin, VitB6, VitB12, VitC, folic acid, VitD, calcium (plus other MN)	*n = 117* non-fortified biscuits plus deworming (Albendazole)	Hb, anemia, ferritin, anthropometrics (HAZ-score; WAZ-score; WHZ-score), function (cognitive; physical), morbidity	2x2 factorial design with 4 groups (total N = 510); here data extracted for (fortified biscuits+deworming) vs deworming (n = 254); some data from additional publication [54] Sponsors: Neys-van Hoogstraten Foundation, The Netherlands; Ellison Medical Foundation
Author, year: Nugroho, 2010 [36] Design: cluster RCT Follow-up (years): 0.42 Sponsor: industry funding	Country: Indonesia Setting: school feeding intervention Target population: children from poor urban elementary school Age (mean):8 years (range: 7 to 9) Males (%): 52	*n = 113* iron & zinc-fortified milk; dual MN strategy MN applied: iron, zink	*n = 105* un-fortified milk	function (cognitive; physical)	Sponsors: Nestle Indonesia
Author, year: Rahman, 2015 [31] Design: cluster RCT Follow-up (years): 0.42 Sponsor: public funding	Country: Bangladesh Setting: community household setting Target population: children from 44 selected rural baris Age (mean):10.4 years (range: 6 to 15) Males (%): 51	*n = 191* iron & VitA-fortified chapatis (made from centrally fortified wheat flour); multi MN strategy MN applied: iron, zink, VitA (retinol palmitate), VitB1, VitB2, niacin, folic acid	*n = 143* un-fortified chapatis (made from non-fortified wheat flour)	Hb, anemia, ferritin	Sponsors: MOST project and USAID Cooperation Agreement
Author, year: Rohner, 2010 [47] Design: RCT Follow-up (years): 0.42 Sponsor: public & industry funding	Country: Cote d’Ivoire Setting: school feeding intervention Target population: school children Age (mean):9.3 years (range: 6 to 14) Males (%): 58	*n = 69* iron-fortified biscuit (FPP); single MN strategy MN applied: iron	*n = 70* non-fortified biscuit (PPP)	Hb, anemia, ferritin, morbidity	2x2x2 factorial design with 8 groups (n = 591); here data extracted for PPP vs FPP (n = 147); some data extracted from additional publication [55] Sponsors: Medicor Foundation; Swiss Foundation for Research in Nutrition; Hochstrasser Foundation; Midor AG; Dafra Pharma; Dr. Lohmann GmbH; Nestle (Abidjan)
Author, year: Rohner, 2010 [47] Design: RCT Follow-up (years): 0.42 Sponsor: public & industry funding	Country: Cote d’Ivoire Setting: school feeding intervention Target population: school children Age (mean):9.9 years (range: 6 to 14) Males (%): 58	*n = 70* iron-fortified biscuit (plus deworming; FPH); single MN strategy MN applied: iron	*n = 65* non-fortified biscuit (plus deworming; PPH)	Hb, anemia, ferritin, morbidity	2x2x2 factorial design with 8 groups (n = 591); here data extracted for PPH vs FPH (n = 143); some data extracted from additional publication [55] Sponsors: Medicor Foundation; Swiss Foundation for Research in Nutrition; Hochstrasser Foundation; Midor AG; Dafra Pharma; Dr. Lohmann GmbH; Nestle (Abidjan)
Author, year: Rohner, 2010 [47] Design: RCT Follow-up (years): 0.42 Sponsor: public & industry funding	Country: Cote d’Ivoire Setting: school feeding intervention Target population: school children Age (mean):10.1 years (range: 6 to 14) Males (%): 57	*n = 76* iron-fortified biscuit (plus malaria therapy; FMP); single MN strategy MN applied: iron	*n = 70* non-fortified biscuit (plus malaria therapy; PMP)	Hb, anemia, ferritin, morbidity	2x2x2 factorial design with 8 groups (n = 591); here data extracted for PMP vs FMP (n = 143); some data extracted from additional publication [55] Sponsors: Medicor Foundation; Swiss Foundation for Research in Nutrition; Hochstrasser Foundation; Midor AG; Dafra Pharma; Dr. Lohmann GmbH; Nestle (Abidjan)
Author, year: Rohner, 2010 [47] Design: RCT Follow-up (years): 0.42 Sponsor: public & industry funding	Country: Cote d’Ivoire Setting: school feeding intervention Target population: school children Age (mean):9.8 years (range: 6 to 14) Males (%): 56	*n = 62* iron-fortified biscuit (plus deworming plus malaria therapy; FMH); single MN strategy MN applied: iron	*n = 72* non-fortified biscuit (plus deworming plus malaria therapy; PMH)	Hb, anemia, ferritin, morbidity	2x2x2 factorial design with 8 groups (n = 591); here data extracted for PMH vs FMH (n = 147); some data extracted from additional publication [55] Sponsors: Medicor Foundation; Swiss Foundation for Research in Nutrition; Hochstrasser Foundation; Midor AG; Dafra Pharma; Dr. Lohmann GmbH; Nestle (Abidjan)
Author, year: Sazawal, 2013 [39] Design: RCT Follow-up (years): 0.42 Sponsor: public & industry funding	Country: Bangladesh Setting: school feeding intervention Target population: children from primary schools Age (mean):7 years (range: 6 to 9) Males (%): 43	*n = 278* MMN fortified yoghurt; multi MN strategy MN applied: iron, zink, iodine, VitA	*n = 293* non-fortified yoghurt	Hb, anemia, ferritin, anthropometrics (height change; weight change; HAZ-score; WAZ-score)	Sponsors: GAIN- Global Alliance for Improved Nutrition; Danone Foods
Author, year: Solon, 2000 [46] Design: RCT Follow-up (years): 0.42 Sponsor: public funding	Country: Philippines Setting: school feeding intervention Target population: children from 4 study schools Age (mean):9.5 years (range: 6 to 13) Males (%): 53	*n = 382* VitA-fortified wheat-flour bun (pandesal); single MN strategy MN applied: VitA	*n = 426* un-fortified wheat-flour bun (pandesal)	Hb, anemia	Sponsors: Johns Hopkins University; The Nutrition Center of the Philippines; Helen Keller International; US Agency for International Development
Author, year: Trinidad, 2014 [27] Design: RCT Follow-up (years): 0.42 Sponsor: industry funding	Country: Philippines Setting: school feeding intervention Target population: children from 6 study schools Age (mean):6 years (range: 6 to no info) Males (%): 0	*n = 84* MMN fortified milk; multi MN strategy MN applied: iron, zink, VitA, VtiD, VitC	*n = 40* water	Hb, ferritin, anthropometrics (height change; weight change)	3 arm trial Sponsors: Nestle Philippines
Author, year: van Stuijvenberg, 2006 [25] Design: RCT Follow-up (years): 0.42 Sponsor: partly industry funding	Country: Republic of South Africa Setting: school feeding intervention Target population: children from low-socio-economic status community school Age (mean):7.9 years (range: 6 to 11) Males (%): 53	*n = 102* iron fortified bread; multi MN strategy MN applied: iron, zinc, VitA, VitB-complex, folic acid	*n = 51* non-fortified bread	Hb, anemia, ferritin	3 arm trial Sponsors: CELANEM and Albion Laboratories
Author, year: van Stuijvenberg, 2008 [26] Design: RCT Follow-up (years): 0.42 Sponsor: partly industry funding	Country: Republic of South Africa Setting: school feeding intervention Target population: school children from low-socio-economic status community Age (mean):9.2 years (range: 6 to 11) Males (%): 51	*n = 257* fortified bread with added iron; single MN strategy MN applied: iron	*n = 82* fortified bread without added iron	Hb, anemia, ferritin	4 arm trial Sponsors: Janssen-Cilag; Akzo Nobel Functional Chemicals
Author, year: van Stuijvenberg, 1999 [45] Design: RCT Follow-up (years): 0.42 Sponsor: industry funding	Country: Republic of South Africa Setting: school feeding intervention Target population: children from rural mountainous area, low socioeconomic status Age (mean):8.7 years (range: 6 to 11) Males (%): 52	*n = 115* MMN fortified biscuits; multi MN strategy MN applied: iron, iodine, beta-carotene, VitC	*n = 110* non-fortified biscuits	Hb, anemia, ferritin, anthropometrics, function (cognitive; physical)	Sponsors: SASKO Ltd; Smith-Kline Beecham Pharmaceuticals
Author, year: Wang, 2017 [44] Design: cluster RCT Follow-up (years): 0.42 Sponsor: public funding	Country: China Setting: school feeding intervention Target population: middle school students Age (mean):13.3 years (range: 12 to 14) Males (%): 27	*n = 137* MN fortified milk; multi MN strategy MN applied: VitA, VitD, VitE, VitB2, phosphorus, zink	*n = 159* non-fortified milk	performance in academic subjects; self-efficacy; cognitive strategies; test anxiety	Sponsors: National Natural Science Foundation of China; China Medical Board
Author, year: Zhang, 2014 [40] Design: RCT Follow-up (years): 0.42 Sponsor: public funding	Country: China Setting: school feeding intervention Target population: children from secondary schools Age (mean):12.9 years (range: 12 to 14) Males (%): 49	*n = 66* High calcium fortified milk powder + VitD; dual MN strategy MN applied: calcium, VitD	*n = 55* low calcium milk powder + VitD	anthropometrics (height change; weight change)	3 arm trial (data extracted for High-Ca group vs. Low-Ca group, not for Mid-Ca group) Sponsors: Key Programs for Science and Technology Development of China; National Natural Science Foundation of China

24 included primary studies are sorted for author name; all 30 pair-wise comparisons are displayed.

Abbreviations: RCT, randomised controlled trials; Hb, haemoglobin; IDA, iron deficiency anaemia.

Most participants were recruited from urban or rural school settings, often with a low socio-economic background. School feeding interventions were the by far most frequent distribution channel of fortified food in this population (22 of 24 studies). Two of 24 studies used a community household setting [28, 31]. The most frequent exclusion criteria were severe anaemia, acute or chronic disease or severe mal- or under-nutrition. The mean age of participants at inclusion was 8.8 (SD 1.7) years (range: 6–13) and the gender ratio was balanced (mean rate of male participants: 51%; median 51 [IQR: 49 to 53]). Mean haemoglobin values at the baseline varied between 10.8 g/dl and 13.0 g/dl across studies (median of study values: 11.7 g/dl). Iron deficiency rates at the baseline, as defined by study authors via ferritin values, were quite low (mean: 21% [SD: 17.6]; range: 1.2% to 63.5%; ten studies with data). Ferritin values were often not adjusted for inflammation. Thus, without further adjustment for inflammation, it cannot be excluded that the prevalence of low iron stores may be higher [56].

Fortified milk was prepared with centrally processed fortified milk or milk powder in most of the studies, while one study used fortified yoghurt [39]. Fortified cereals were most often in the form of biscuits, cookies, bread, maize porridge or chapattis. A variety of MNs were used for fortification. Iron was the most frequent MN: 17 of 24 trials applied iron either as a single MN fortification (six studies) or in combination with other MNs, e.g. zinc, iodine, vitamin B, vitamin C, folic acid, calcium or vitamin D (two studies as dual and nine studies as MMN fortification). The mean difference between the intervention and control group in iron consumed per day was 8.3 mg (median: 7.1 mg; IQR: 5.5–11.4). The seven of studies 24 studies without iron fortification applied either a single MN strategy (with vitamin A [46]; calcium [43]; vitamin D [42]), a dual MN strategy (calcium plus vitamin D [37, 38, 40]), or a MMN strategy (vitamins A, D, E, B2; phosphorus; zink [44]). For our analysis, we extracted 30 pair-wise comparisons for MN interventions in 7568 children and adolescents, as several studies with more than two intervention and control groups contributed several independent comparisons (i.e. with no intervention or control group in common) [18]. In our results section, we have relied on these 30 pair-wise comparisons as the unit of analysis.

### Risk of bias and confidence in cumulative evidence

If a study described an adequate method in a specific risk of bias domain (e.g. adequate blinding of outcome assessment), it was judged as “low risk of bias” in this domain. Description of an in-adequate method was judged as “high risk of bias” and, if incomplete information was given, as “unclear risk of bias”.

Only 4 of 24 studies provided enough information to conclude that both random sequence generation and allocation concealment was adequately performed (Table 3). Adequate blinding of participants and personnel was reported in 17 of 24 studies and adequate blinding of outcome assessment in 15 of 24 studies. Incomplete outcome data were addressed in 19 of 24 trials. For three of 24 studies a study protocol was available to judge possible reporting bias [29, 42, 47]. In two of these three studies, outcome reporting was not complete [42, 47] and only one of 24 trials was judged as having a low risk of reporting bias [29]. Finally, only 4 of 24 studies were judged as having a low risk of bias in at least 5 of 6 assessed domains (two studies with iron fortification [24, 26]; two with calcium and/or vitamin D fortification [40, 42]). An assessment of bias across studies (publication bias) for haemoglobin change was done with a funnel plot (S1 Fig). Visual inspection of the funnel-plot showed no asymmetry and was interpreted as not suspicious for small study effects (Egger’s test: p = 0.44).

**Table 3:** Risk of bias summary table.

author	year	Random sequence generation (selection bias)	Allocation conceal-ment (selection bias)	Blinding of partici-pants and personel (perfor-mance bias)	Blinding of outcome assess-ment (detection bias)	Incomplete outcome data (attrition bias)	Selective reporting (reporting bias)
Andang’o	2007	**+**	**+**	**+**	**+**	**+**	**-**
Bardosono	2009	**-**	**?**	**+**	**+**	**+**	**?**
Bass	2007	**+**	**?**	**+**	**+**	**+**	**?**
Bouhouch	2016	**+**	**?**	**+**	**?**	**+**	**+**
Camargo	2012	**+**	**+**	**+**	**+**	**+**	**-**
Du	2004	**?**	**?**	**?**	**?**	**+**	**?**
Hieu	2012	**+**	**?**	**+**	**+**	**-**	**?**
Kuriyan	2016	**+**	**?**	**+**	**+**	**+**	**?**
Le	2007	**?**	**+**	**+**	**?**	**+**	**?**
Muthayya	2012	**+**	**?**	**+**	**+**	**+**	**?**
Nestel	2004	**?**	**?**	**+**	**+**	**-**	**-**
Neyestani	2013	**?**	**?**	**+**	**+**	**+**	**?**
Nga	2011	**+**	**?**	**+**	**+**	**+**	**?**
Nugroho	2010	**?**	**?**	**?**	**?**	**?**	**-**
Rahman	2015	**+**	**?**	**+**	**+**	**+**	**-**
Rohner	2010	**?**	**?**	**+**	**+**	**+**	**-**
Sazawal	2013	**+**	**?**	**+**	**+**	**-**	**-**
Solon	2000	**?**	**?**	**?**	**?**	**+**	**-**
Trinidad	2014	**?**	**?**	**?**	**?**	**+**	**-**
van Stuijvenberg	2006	**+**	**?**	**-**	**-**	**+**	**-**
van Stuijvenberg	2008	**+**	**+**	**+**	**+**	**+**	**-**
van Stuijvenberg	1999	**-**	**?**	**-**	**-**	**+**	**?**
Wang	2017	**+**	**?**	**-**	**-**	**-**	**-**
Zhang	2014	**+**	**+**	**+**	**+**	**+**	**?**

The table presents 24 studies by assessed source of bias in a cross-tabulation. Studies are sorted alphabetically by author’s name.

Coding of judgements: “+”: Low risk of bias (adequate method described in this risk of bias domain); “-“: High risk of bias (in-adequate method described); “?”: Unclear risk of bias (incomplete information was given)”

Using GRADE (Table 4), we rated the quality of evidence for the improvement of the haematological outcomes haemoglobin, anaemia, iron deficiency anaemia and iron deficiency as very low (because of the risk of bias, inconsistency and imprecision). The quality of evidence for improvement of anthropometric measures was rated as low due to the risk of bias and imprecision. The quality of evidence for improvement of cognitive and functional measures, physical performance and morbidity was rated as very low due to the risk of bias and inconsistency.

**Table 4:** GRADE evidence profile. Fortified dairy products and cereal food compared with no fortification in MN deficient schoolchildren and adolescents (Question: What is the health impact of fortified dairy products and cereal food on schoolchildren and adolescents (5–15 years)? Setting: Community).

Quality assessment							№ of participants		Effect		Quality	Comment
№ of studies	Study design	Risk of bias	Inconsistency	Indirectness	Imprecision	Other considerations	fortified dairy products and cereal food	no fortification	Relative (95% CI)	Absolute (95% CI)		
**Haemoglobin** (follow up: mean 8 months; assessed with: blood level (g/dl))												
14	randomised trials	serious ^a^	serious ^b^	not serious	serious ^c^	none	2790	2065	-	MD **0.09 g/dl higher** (0.01 lower to 0.18 higher)	⨁◯◯◯ VERY LOW Due to risk of bias, inconsistency and imprecision	A haemoglobin change of 0.09 g/dl may not be clinically important.
**Anaemia** (follow up: mean 8 months; assessed with anaemia rates)												
12	randomised trials	serious ^a^	serious ^b^	not serious	serious ^c^	none	612/2410 (25.4%)	537/1827 (29.4%)	**RR 0.87** (0.76 to 1.01)	**38 fewer per 1'000** (from 3 more to 71 fewer)	⨁◯◯◯ VERY LOW Due to risk of bias, inconsistency and imprecision	
**Iron deficiency anaemia** (follow up: mean 8 months; assessed with iron deficiency anaemia rates [i.e. anaemia in the presence of iron deficiency])												
5	randomised trials	Very serious ^a^	serious ^b^	not serious	not serious	reporting bias suspected	65/1010 (6.4%)	83/647 (12.8%)	**RR 0.38** (0.18 to 0.81)	**79 fewer per 1'000** (from 24 fewer to 105 fewer)	⨁◯◯◯ VERY LOW Due to risk of bias and inconsistency	
**Iron deficiency** (follow up: mean 8 months; assessed with serum ferritin level)												
8	randomised trials	Very serious ^a^	serious ^b^	not serious	not serious	reporting bias suspected	210/1539 (13.7%)	309/1138 (27.2%)	**RR 0.62** (0.40 to 0.97)	**103 fewer per 1'000** (from 8 fewer to 163 fewer)	⨁◯◯◯ VERY LOW Due to risk of bias and inconsistency	
**Anthropometrics** (follow up: mean 8 months; assessed with: HAZ-score: stunting)												
3	randomised trials	serious ^a^	not serious	not serious	serious ^c^	none	571	590	-	MD **0.022 SD higher** (0.069 lower to 0.122 higher)	⨁⨁◯◯ LOW Due to risk of bias, and imprecision	There may be little or no difference in stunting (median HAZ was -1.52 at baseline) ^d^
**Cognitive and functional measures** (follow up: mean 8 months; assessed with: validated test batteries)												
8	randomised trials	serious ^a^	serious ^b^	not serious	(no meta-analysis) ^c^	No information about minimal important difference was given.	Four of 8 studies reported significantly better results for the fortification groups (e.g. improved cognitive performance and working memory, improved problem solving, better short term memory and attention span). Four of 8 studies did not find cognitive or functional improvements.				⨁◯◯◯ VERY LOW Due to risk of bias, inconsistency and unclear minimal important difference	
**Physical performance** (follow up: mean 5 months; assessed with: validated test batteries)												
2	randomised trials	serious ^a^	serious ^b^	not serious	(no meta-analysis) ^c^	No information about minimal important difference was given.	Two studies assessed physical performance and showed no improvement related to food fortification (applied tests: modified Harvard step test; Illinois agility test, shuttle test).				⨁◯◯◯ VERY LOW Due to risk of bias, inconsistency and unclear minimal important difference	
**Morbidity** (follow up: mean 8 months; assessed with: days with illness; risk of illness)												
6	randomised trials	serious ^a^	Very serious ^b^	not serious	(no meta-analysis) ^c^	none	Three of 6 studies reported improved health status for the fortification groups (less days with respiratory-related and diarrhea-related illnesses; reduced risk of acute respiratory infections; protective effect of iron MMN fortification on intestinal parasite infection). Three of 6 studies found no effect on malaria or intestinal parasite infection or diarrhea, vomiting and fever.				⨁◯◯◯ VERY LOW Due to risk of bias and inconsistency	
**Adverse events** (follow up: mean 4 months; assessed with: days with illness; risk of illness)												
3	randomised trials	serious ^a^	serious ^b^	not serious	(no meta-analysis) ^c^	Reporting bias very likely	Three of 24 studies concluded that no significant adverse events were related to the study food or to the fortification				⨁⨁◯◯ LOW Due to risk of bias and inconsistency	

**Abbreviations: CI:** Confidence interval; **MD:** (weighted) Mean difference; **RR:** Risk ratio

^a^. **Haemoglobin**: unclear risk of selection bias (allocation concealment) in 11 of 14 RCT; **Anaemia**: unclear risk of selection bias (random sequence generation: 5 of 12 RCT; allocation concealment: 10 of 12 RCT); **Iron deficiency anaemia**: unclear risk of selection bias (allocation concealment) in 4 of 5 RCT; unclear or high risk of reporting bias (selective reporting) in 4 of 5 studies **Iron deficiency**: unclear risk of selection bias (allocation concealment) in 7 of 8 RCT; unclear or high risk of reporting bias (selective reporting) in 7 of 8 studies. **Anthropometrics (HAZ-score):** unclear risk of selection bias (allocation concealment) in 3 of 3 RCT; high risk of attrition bias (incomplete outcome data) in 1 of 3 RCT **Cognitive and functional measures:** high or unclear risk of selection bias (random sequence generation: 3 of 8 RCT; allocation concealment: 8 of 8 RCT); high or unclear risk of detection bias (blinding of outcome assessment): 4 of 8 RCT; **Physical performance:** unclear risk of selection bias (allocation concealment) in 2 of 2 RCT**; Morbidity**: high or unclear risk of selection bias (random sequence generation: 3 of 6 RCT; allocation concealment: 4 of 6 RCT); **Adverse events**: Reporting bias very likely with only 3 of 24 studies reporting about possible adverse events

^b^. **Haemoglobin**: Unexplained heterogeneity; I-square: 67.7%; **Anaemia**: Unexplained heterogeneity; I-square: 70.7%; **Iron deficiency anaemia**: Unexplained heterogeneity; I-square: 69.5%; **Iron deficiency**: Unexplained heterogeneity; I-square: 82.9%; **Cognitive and functional measures**: high variability in applied tests and outcomes; high variability in results (4 of 8 studies with improvement; 4 of 8 studies showed no effect); **Physical performance**: variability in applied tests and outcomes in a limited number of studies; **Morbidity**: heterogeneity of outcomes for morbidity; high variability in results (3 of 6 studies with improved health status; 3 of 6 studies showed no effect on health status); **Adverse events**: heterogeneity in reported outcomes in a very limited number of studies

^c^. **Haemoglobin**: CI inlcudes both benefit and harm; **Anaemia**: CI inlcudes both benefit and harm; **Anthropometrics (HAZ-score):** CI inlcudes both benefit and harm; **Cognitive and functional measures, Physical performance, Morbidity, Adverse events:** no meta-analysis performed

^d^. **Anthropometrics**: Median HAZ was -1.52 at baseline; Also for WHZ-score (wasting) no significant change emerged (MD: 0.02: 95%-CI: -0.12 to 0.15; 1 study), as well as for (simple) height change and weight change.

### Effect on haemoglobin levels

The effect of fortification on haematological outcomes (haemoglobin levels, anaemia, iron deficiency anaemia, iron deficiency) was assessed in the meta-analysis with 14 studies reporting iron fortification, applied as single [28, 45, 47, 49, 51], dual (i.e. with zinc) [35] or MMN strategy (e.g. with zinc, iodine, vitamin A [24, 25, 27, 31, 39, 41, 50].

**Fig 2:**
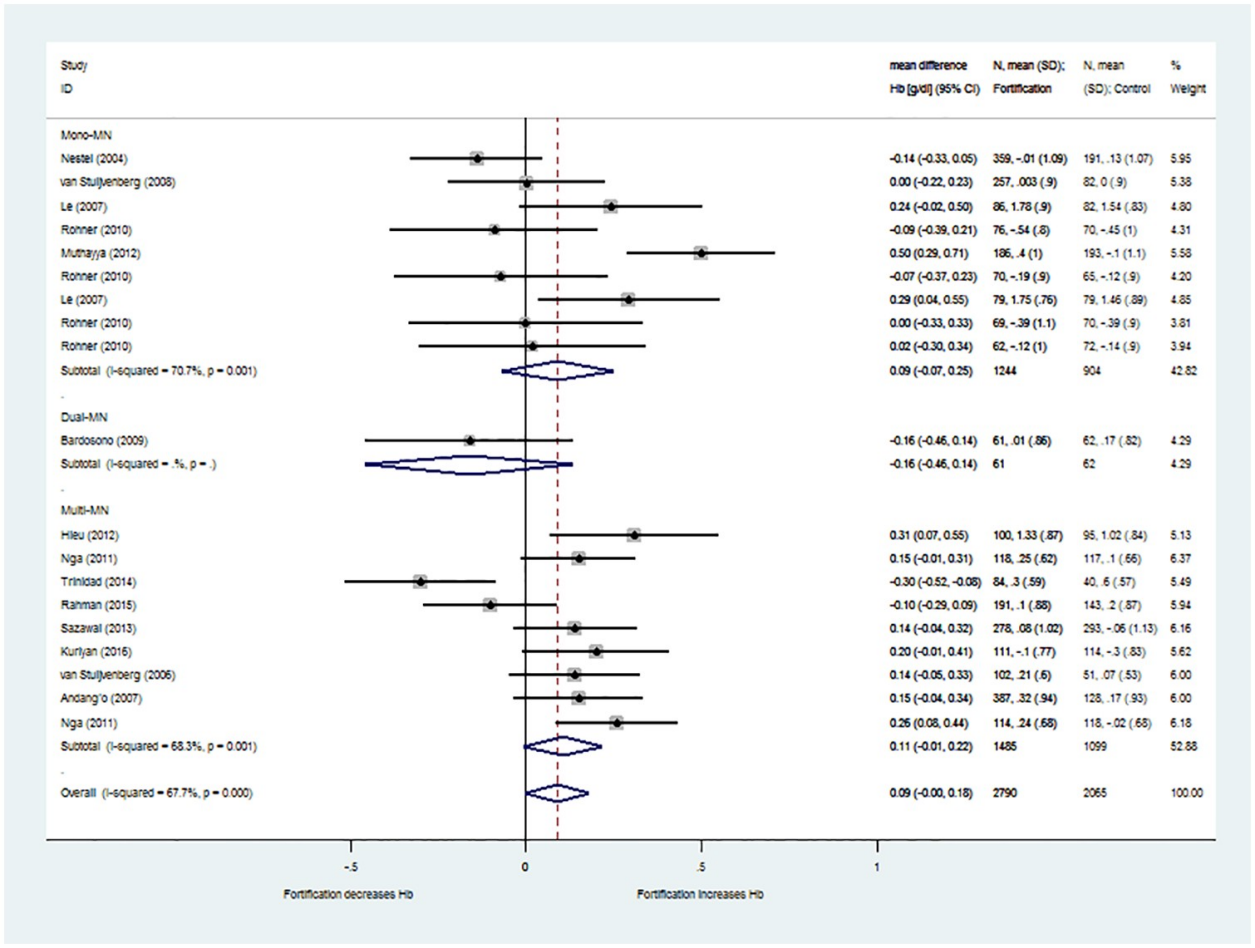
Effect of iron fortified dairy products and cereals on haemoglobin (Hb) levels compared with non-fortified food. Studies with iron fortification included (n = 14 RCT with 19 pair-wise comparisons). Results are provided as weighted mean difference in haemoglobin (WMD: g/dl with 95%-CI; conversion to g/L with factor 10) between intervention and control group.

Haemoglobin blood level was the most frequently reported outcome parameter. Iron fortification led to a very small and non-significant increase of haemoglobin compared with the control group (0.09 g/dl; 95%-CI: -0.01 to 0.18; I^2^ = 71%; Fig 2). No statistically significant or clinically relevant effect on haemoglobin change was found for two assessed pre-specified subgroups (fortified dairy products vs. cereals; single vs. dual vs. MMN strategy).

One study with single MN vitamin A fortification of wheat flour without iron [46] improved vitamin A status, but the haemoglobin level remained basically unchanged (-0.03 g/dl; 95%-CI: -0.21 to 0.15).

### Effects on anaemia prevalence

**Fig 3:**
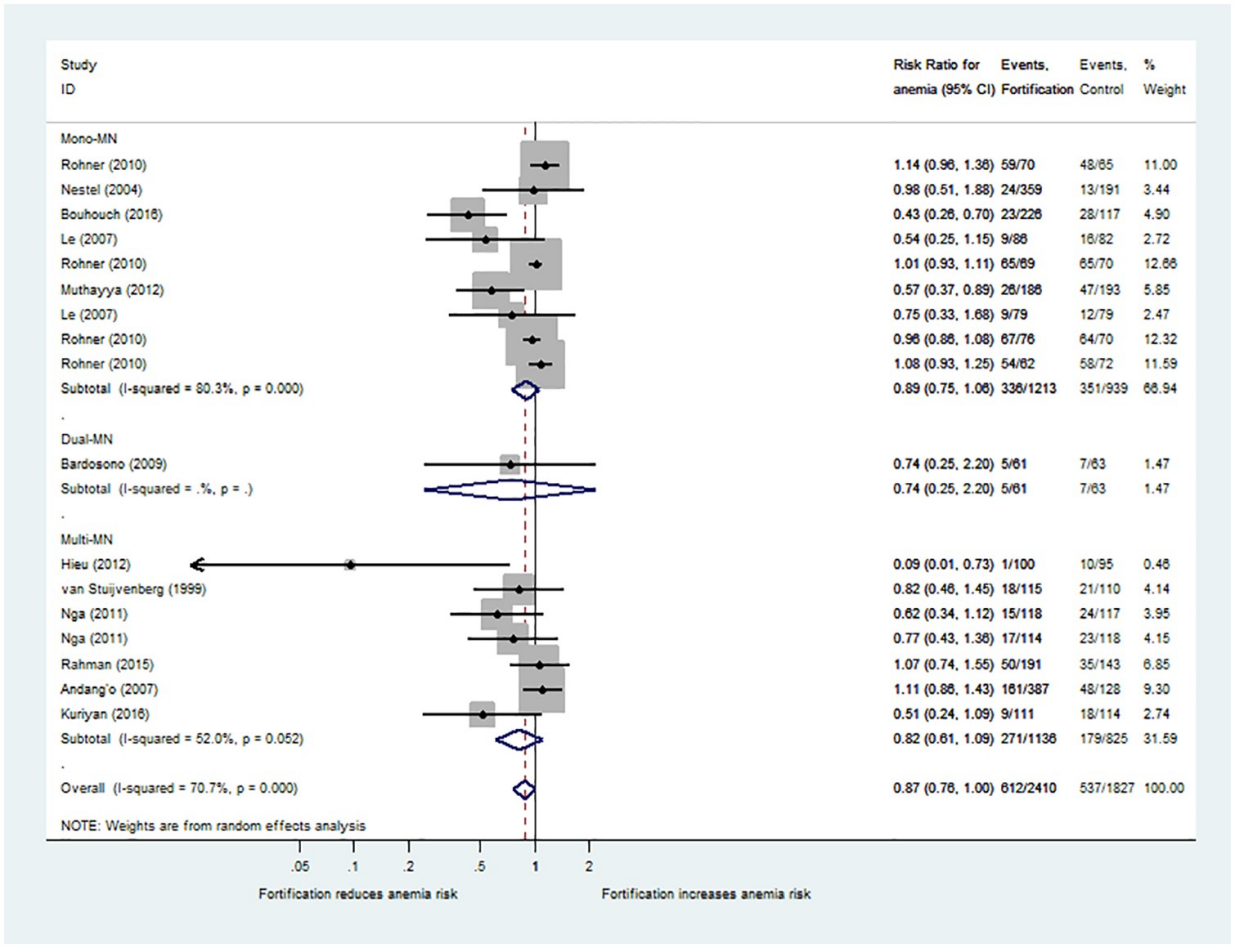
Effect of iron-fortified dairy products and cereals on anaemia compared with non-fortified food. Studies with iron fortification included (n = 12 RCT with 17 pair-wise comparisons). Results are provided as risk ratio (RR, 95%-CI) of suffering from anaemia in the intervention group compared with the control group.

Twelve studies with iron fortification, often in combination with other MN, provided data for 17 pair-wise comparisons of anaemia rates [24, 28, 29, 31, 35, 41, 45, 47–51]. In general, authors defined anaemia using thresholds for mild anaemia according to WHO recommendations (<11.5 g/dl [5–11 years of age]; <12.0 g/dl [12–15 years of age]). The mean anaemia rate across studies at the baseline was 44% (median: 35%; IQR: 20%-71%; 16 comparisons with data). Based on the available data, iron-fortified dairy products or cereals did not significantly reduce the risk of suffering from anaemia in the main analysis (risk ratio 0.87; 95%-CI: 0.76 to 1.01; I^2^ = 71%; Fig 3).

**Fig 4:**
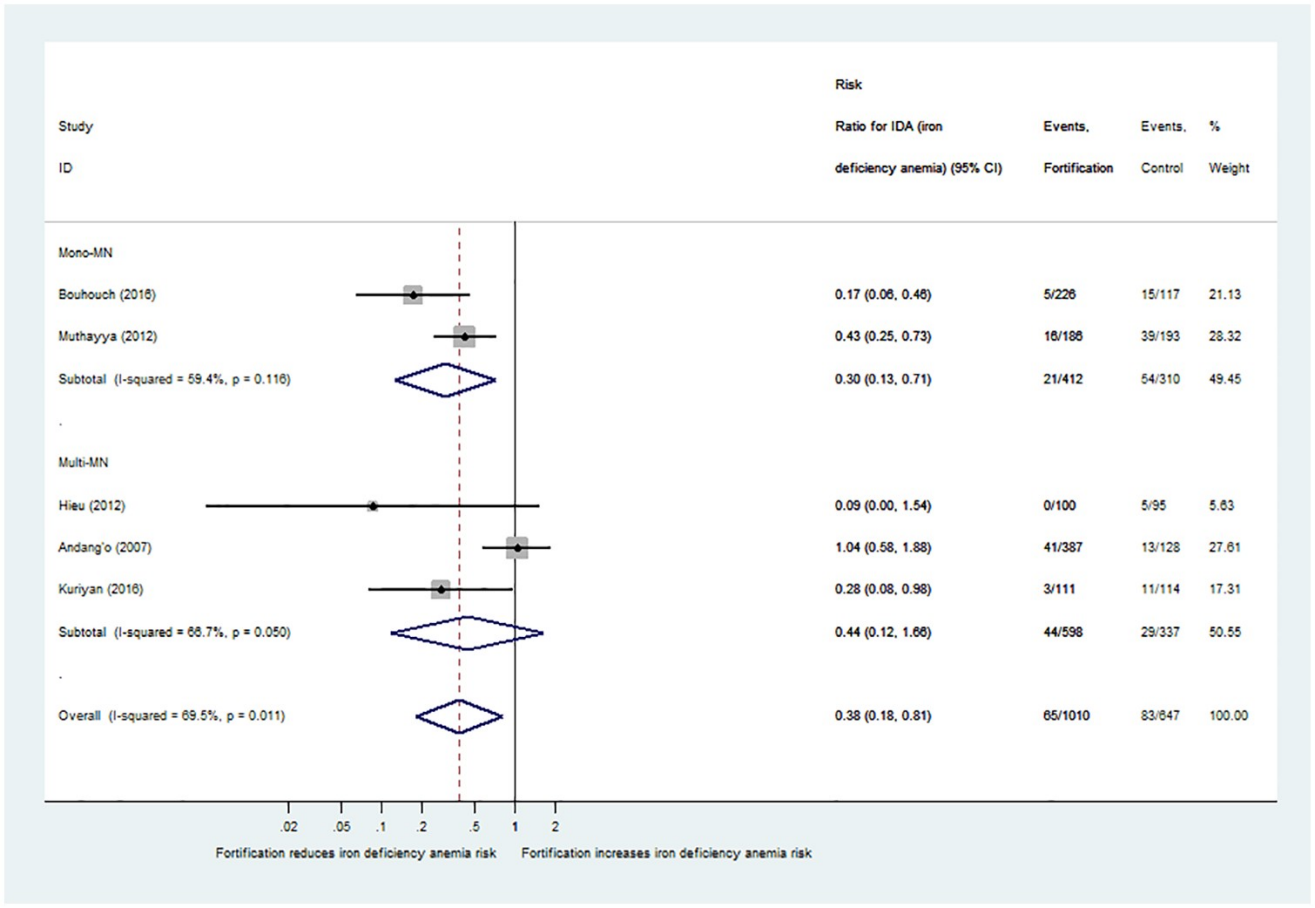
Effect of iron-fortified dairy products and cereals on iron deficiency anaemia compared with non-fortified food. Studies with iron fortification included (n = 5 RCT with 5 pair-wise comparisons). Results are provided as risk ratio (RR, 95%-CI) of suffering from iron deficiency anaemia in the intervention group compared with the control group.

The risk of iron deficiency anaemia decreased with iron fortification (risk ratio 0.38; 95%-CI: 0.18 to 0.81), but only 5 comparisons provided data [24, 29, 41, 49, 50] and underreporting may have biased the results (Fig 4).

Furthermore, iron deficiency rates decreased with iron fortification (risk ratio 0.62; 95%-CI: 0.40 to 0.97; 8 studies with 11 comparisons [24, 29, 31, 35, 41, 47, 49, 50]; S2 Fig). However, distribution of medians of ferritin levels at the end of study was similar in the intervention and control groups (ranges of ferritin medians at the end of study [micro-g/l]: intervention: 21.4 to 68.3; control: 14.5 to 69.3; 11 of 18 comparisons with data). Five studies [25, 27, 31, 35, 39] provided mean values and showed a very small non-significant increase in ferritin levels with iron fortification (0.52 micro-g/l; 95%-CI: -0.93 to 1.97; I^2^ = 0%). No significant effect on anaemia risk was found for any of the assessed pre-specified subgroups (fortified dairy products vs. cereals; single vs. dual vs. MMN strategy).

The study with single MN vitamin A fortification of wheat flour without iron [46] lead to a non-significant increase in anaemia risk (risk ratio 1.29; 95%-CI: 0.93 to 1.77).

### Effects on anthropometric measures

These outcomes were reported by studies with iron fortification (often in combination with zinc, iodine, vitamin A and other MN), as well as by studies using calcium, vitamin D or vitamin A without iron. At the baseline, the prevalence of stunting (HAZ-score) and wasting (WHZ-score), each defined as z-scores < -2 SD, was less than 25% across studies (median HAZ -1.52, IQR: -1.75 to -1.39; eight studies with data; median WHZ: -0.86, IQR: -1.07 to -0.75; six studies).

In our meta-analysis, no significant increase of z-scores emerged for HAZ- or WHZ-scores (HAZ: 0.02; 95%-CI:—0.07 to 0.11; four comparisons with data; WHZ: 0.02; 95%-CI:—0.12 to 0.15; one study). Some studies also reported direct measures for height or weight gain with a broad range across studies, depending on the age of participants and length of follow up. No significant difference emerged for height change and weight change between intervention groups and control groups (mean height change [cm]: 4.7 vs. 4.7; p = 0.66; mean weight change [kg]: 3.33 vs. 2.96; p = 0.07). Results were similar, when the analysis was restricted to the five studies with vitamin D and/or calcium fortification [37, 38, 40, 42, 43] or to the only retrieved study from a high income country (Australia) [43] in our review.

### Cognitive development, functional measures and morbidity

These outcomes were reported by studies using dual and multi MN strategies (often iron fortification in combination with zinc, iodine, vitamin A and other MN). Eight studies provided information about cognitive tests and functional measures [29, 35, 36, 41, 44, 45, 48, 49]. Here we report only on results based on validated cognitive test batteries and not on anecdotal information about cognitive performance to increase validity of results. We have not performed meta-analysis for cognitive and functional measures, as we judged the available data as not suitable for meta-analyses due to methodological heterogeneity (for example, a broad range of different tests was used to measure cognitive performance).

Four of these eight studies (mean follow-up: six months) reported significantly better results for the fortification groups using validated cognitive test batteries. For example, improved cognitive performance and working memory (applied test: cognitive assessment battery, including coding score, digit-span forward, digit-span backward and visual search [35]), improved cognitive strategies and problem solving (applied test: Motivated Strategies for Learning Questionnaire (MLSQ) [44]), as well as better short-term memory and attention span were found (applied test: Wechsler Intelligence Scales III [36, 48]). Magnitudes of the minimal important difference for each of the applied cognitive tests were not reported.

Four of eight studies with equivalent follow-up length did not find improvements in cognitive tests (applied tests: Kaufman Assessment Battery for Children, KABC-II [29]; battery of cognitive tests assessing mental alertness, short term memory and executive functions [41]; battery of tests assessing short term memory and retrieval ability, cognitive speed, fluid reasoning [49]; cognitive test battery assessing speed of processing and capacity of work memory [45]). Two of eight studies assessed physical performance and showed no improvement related to food fortification (applied tests: modified Harvard step test, [35]; Illinois agility test, shuttle test [41]).

Six studies provided information about morbidity [41, 42, 45, 47, 48, 51]. Three of these six studies reported improved health status for the fortification groups. In one study from South Africa using iron MMN fortification [45], children in the fortification group had fewer days with respiratory-related illnesses (33 days vs. 47 days/100 children; p = 0.097) and diarrhoea-related illnesses (52 days vs. 79 days/100 children, p = 0.013). In one study from Mongolia on vitamin D fortification [42], the risk of acute respiratory infections in the intervention group was reduced (risk ratio 0.5; 95%-CI: 0.28 to 0.88). One four-arm study from Vietnam [48], which also applied deworming in two arms found a significant protective effect of iron MMN fortification on intestinal parasite infection with Ascaris (RR 0.30; 95% CI = 0.15–0.59) and Trichuris (RR 0.36; 95% CI = 0.18–0.73). Three of the six studies with morbidity data found no effect of fortification on malaria or intestinal parasite infection rates [47, 51], or on diarrhoea, vomiting and fever [41].

Only three out of 24 studies reported on adverse events monitored during the trial. These studies concluded that no significant adverse events were related to the study food or to the fortification [29, 41, 42]. Two studies reported on acceptability of fortified food (acceptance: 85% of children with fortified milk declared having no problem with taste [38]; adherence of 73% to fortified milk in the intervention group [40]). We found no evidence on the pre-specified outcomes quality of life and mortality.

### Exploring heterogeneity

In our multivariable meta-regression analysis, none of the independent variables was significantly associated with a change in haemoglobin (17 of 19 pairwise comparisons with data; pre-specified variables: mean haemoglobin level at the start of the study (p = 0.59); the daily amount of consumed iron (p = 0.87), the length of follow-up (p = 0.37), completeness of follow-up (p = 0.68)). In addition, none of the independent variables was significantly associated with anaemia risk (14 of 17 pairwise comparisons with data; pre-specified variables: mean haemoglobin level at the start of the study (p = 0.44); the daily amount of consumed iron (p = 0.67), the length of follow-up (p = 0.83), completeness of follow-up (p = 0.95)).

We performed a sensitivity analysis for the hematologic outcomes with low risk of bias studies. The two iron fortification studies with low risk of bias in 5 of 6 domains [24, 26] showed similar small effects as found in our main analysis for the hematologic outcomes haemoglobin (changes: 0.15 g/dl [24] and 0.01 g/dl [26]) and iron deficiency risk (RR 0.61 [24]). Risk for iron deficiency anaemia was higher (RR 1.04 [24]) and no data were available for anaemia risk in these two studies. Another sensitivity analysis showed basically unchanged results using mean haemoglobin values of groups at the end of the study instead of mean changes of groups (haemoglobin change: 0.08 g/dl [-0.03 to 0.18], 16 comparisons).

In addition, we assessed, if there was an impact of industry funded studies on overall study results in our data set. We compared industry funded studies (comprising exclusive industry funding, industry funding together with public agencies or no information was available) with non-industry funded studies (i.e. exclusive funding by public agencies or other non-industry funding sources, for example private foundations). No significant difference emerged for results of primary or secondary outcomes between industry and non-industry funded studies (S4 Table).

## Discussion

In our systematic review, we assessed the effects of fortified milk and cereal food with different MNs on direct health measures in 9,367 children and adolescents in the age group 5–15 years, based on data from 24 included studies. Fortification led to a very small and non-significant increase in haemoglobin values in this age group that may not be clinically relevant (very low quality of evidence).

We are not aware of any publication that has defined the clinically important difference in haemoglobin values for physical and cognitive development in children and adolescents. WHO defines the range of mild anemia in children (age 5–11 years) from 11.4 to 11.0 g/dl [57]. Thus, a haemoglobin change of about 0.4 g/dl would allow a 5–11 year old child to move from “mild anemia” to “non-anemia”. This magnitude may be seen as a proxy for a minimal important difference. An increase of about 0.1 g/dl as found in our review is well below a difference of 0.4 g/dl.

While no relevant effect on anaemia risk emerged in our main analysis, the prevalence of iron deficiency anaemia decreased, though underreporting may have biased the results. No relevant impact on anthropometric measures was found. However, only some studies provided data and follow-up was short. Very low quality of evidence emerged for the improvement of cognitive performance, functional measures and morbidity.

### Limitations

We performed a thorough search of published evidence using established electronic literature databases, screening of references of relevant systematic reviews and included studies, as well as citation searches of included studies. Nevertheless, we cannot rule out the possibility of having overlooked relevant studies. For example, we did not perform a grey literature search.

Other limitations need to be mentioned. 1. Iron deficiency rates were relatively low in our study population and several studies were performed in malaria endemic zones or with a high rate of infections among participants (e.g. with helminths). This may have attenuated the effect of fortification. 2. The follow-up time for most of the studies was short. Hence, the impact of fortification on some outcome domains (e.g. height and weight gain) may have been underestimated. 3. The risk of bias for the most frequently reported outcomes haemoglobin change and anaemia rates is unclear. The studies conducted often showed methodological shortcomings. 4. Seven of 24 studies were cluster-randomised trials, but only in two cases [31, 42] had authors adjusted results for intra cluster correlation. For our analysis, we accounted for clustering by calculating the effective sample size of trials without adjustment [18]. 5. The full text selection, the data extraction, the risk of bias assessment and GRADE estimations were performed by one reviewer and confirmed by a second reviewer and not performed independently in duplicate. This approach has some limitations and may lead to more errors. 6. The included primary studies in our review have not assessed the MN composition of usual food intake, to check if intakes of the fortified MN apart from the given fortified foods were similar. However, as most of the populations in the RCTs were blinded concerning their group allocation, we have no reason to assume, that there were systematic differences between groups concerning the MN content of usual food. 7. Statistical heterogeneity across trials was substantial and results of the meta-analysis should be interpreted with caution. Heterogeneity remained largely unexplained in our subgroup and meta-regression analyses. Possible explanations include reporting bias with underreported co-interventions (e.g. educational interventions; contamination in non-cluster-randomised trials), variability in baseline iron deficiency, variable adherence to fortified food or the unclear impact of different MN-compounds and MN-dosages used for different carriers. For example, in 26 iron intervention groups, eight groups used electrolytic iron, six groups NaFeEDTA and five groups ferrous fumarate.

### Existing evidence and research needs

The impact of fortified food and cereals on haemoglobin levels and anaemia rates is lower in this age group than in younger children from 6 months to 5 years old. [14, 15] This may be because iron deficiency rates were relatively low in our study population. Other reasons for anaemia, such as subclinical infections (e.g. with malaria, parasites), environmental enteric dysfunction, micronutrient deficiencies other than iron, (i.e. vitamin A, B6, B12, riboflavin, folate, copper) or genetic factors (e.g. haemoglobinopathies) [58], may also have contributed to the modest effect. At the baseline, most of the participants had haemoglobin values on the upper threshold of mild anaemia (Hb IQR: 11.1–12.6 mg/dl). Thus, even a small increase of haemeoglobin might go hand in hand with reduced risk of anaemia and iron deficiency anaemia after intervention.

Several systematic reviews have assessed the impact of fortified food on health outcomes in children and adolescents but a comparison of these results with our findings is not straightforward. For example, one review with 201 included studies [15] across various food carriers, where populations in 8 of 41 studies included had some form of iron deficiency, found increased haemoglobin levels and a reduced anaemia risk in preschool and schoolchildren, but results were often combined for preschool and schoolchildren and also non-randomised before-after studies were included. As in our review, no significant impact on weight and height gain was found. Another review with 60 included studies [12] also reported increased haemoglobin levels, but results were pooled for children and adults. No effect on growth or mental and motor development was found. Another review with 12 included studies and without meta-analysis [59] reported improved haemoglobin concentration, but the baseline iron status of populations remains open and many different food carriers of fortification were included (e.g. beverages, milk, condiments). Impact on morbidity, growth and cognitive measures was deemed to be unclear.

Ultimately, evaluations of large-scale field implementations are important when judging the impact of food fortification in real life. A recently published systematic review with 13 included studies and without meta-analysis [60] compiled the evidence of the effectiveness of implemented flour fortification programmes on iron status and anaemia for several subgroups of children, including schoolchildren. Significant decreases in the prevalence of low ferritin occurred only in 1 of 6 subgroups and in 4 of 13 subgroups for anaemia prevalence. The authors therefore conclude that the evidence for reducing the prevalence of anaemia via large flour fortification programmes is limited in this population group. Centrally-processed, fortified cereal food with potentially higher bioavailability of micronutrients may yield different results, but that was not the focus of this review.

In future research projects, MN fortification with iron should be tailored to anemic population groups with low iron stores to exploit their potential health impact fully. In addition, the optimal iron compound and iron dosage for specific food carriers (e.g. milk; cereals) still have to be defined. Some Cochrane systematic reviews which will assess food fortification for populations (including the age group 5–15 years) are underway, but the protocols focus mainly on single micronutrients such as iron [61, 62], zinc [63], folate [64] or vitamin A [65]. Nevertheless, these reviews will contribute additional knowledge, as they also include non-randomised studies of field evaluations which may have carried out long-term follow-up of study populations.

### Implications for public health decision makers

The results of our systematic review provide important knowledge for policy makers as the full spectrum of different MN and fortification strategies for dairy products and cereal food was directly reviewed in this age group. Several policy recommendations arise: targeted interventions should last over longer periods to uncover possible long-term health effects, for example on anthropometric measures and cognitive development [10].

Continuous monitoring is needed with comprehensive reporting of morbidity and functional measures under real world conditions. Ultimately, this should be conducted through field studies, where the public health effect is evaluated against no intervention. Here, an extra energy and macronutrient effect of fortified food on anthropometric measures can be expected.

Our review was not limited to interventions delivered in schools, but most of the included studies covered this setting. Despite the very low quality of evidence of short-term health effects in our data set, school feeding programmes, as the typical setting for fortified dairy products and cereals in this age group, can have an additional impact on population health in the longer run, which is not represented by the short follow-up time of the randomised trials (e.g. improved cognitive performance). School feeding programs have advantages over other public health approaches. A daily school meal is an incentive for low-income families to send children to school and can result in improved attendance [10]. Thus, school feeding programmes are an essential safety net and an excellent opportunity for additional interventions with proven health benefits in the community setting, for example, thorough nutritional education, information about suitable sanitation measures for clean water, vaccination programmes, malaria prevention and treatment for intestinal parasites [9, 66].

## Conclusions

Fortification of dairy products and cereal food had only marginal health effects in our sample population of children and adolescents from 5–15 years. Further evidence is needed from experimental studies, cohort studies with a longer follow up period and evaluations of large-scale implementation programmes to better understand the health impact of fortified dairy products and cereal food on functional and cognitive development, as well as on morbidity, in this age group.

## Supporting information

S1 Fig Funnel plot. The effect sizes of difference in haemoglobin and the standard errors of the effect size are displayed for 19 pair-wise comparisons from 14 RCT with iron fortification.
(DOCX)

S2 Fig Effect of iron-fortified dairy products and cereals on iron deficiency compared with non-fortified food. Studies with iron fortification included (n = 8 RCT with 11 pair-wise comparisons). Results are provided as risk ratio (RR, 95%-CI) of suffering from iron deficiency in the intervention group compared with the control group. Displayed subgroups: iron single MN fortification (1); iron dual MN fortification (2), iron multi MN fortification (3).
(DOCX)

S1 Table PRISMA checklist. (DOCX)

S2 Table Inclusion and exclusion criteria of the systematic review. (DOCX)

S3 Table Examples of excluded studies. Studies are listed alphabetically by author’s name.
(DOCX)

S4 Table Comparison of results of industry funded studies vs. non-industry funded studies. (DOCX)

S5 Table Risk of bias assessment: Applied criteria. (PDF)

S1 SP Study protocol. (PDF)

S1 Data (XLS)

## Abbreviations

Fe: iron
MN: micronutrient
MNs: micronutrients
MMN: multi-micronutrient
PWC: pair-wise comparison
RCT: randomised controlled trial
WMD: weighted mean difference
