# Supplementary material for: Health effects of micronutrient fortified dairy products and cereal food for children and adolescents: A systematic review
Source: PLoS One. 2019 Jan 23;14(1):e0210899. doi: 10.1371/journal.pone.0210899 (PMC6343890; doi:10.1371/journal.pone.0210899)
Supplement: S1 Fig — The effect sizes of difference in haemoglobin and the standard errors of the effect size are displayed for 19 pair-wise comparisons from 14 RCT with iron fortification. (DOCX) [file pone.0210899.s001.docx]

**S1 Fig**. **Funnel plot.** The effect sizes of difference in haemoglobin and the standard errors of the effect size are displayed for 19 pair-wise comparisons from 14 RCT with iron fortification.


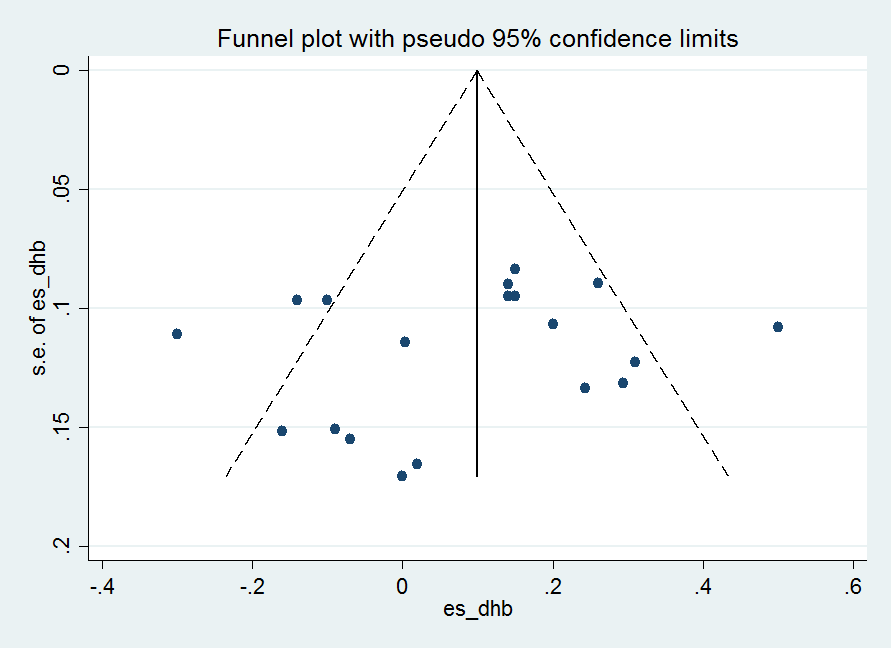


Abbreviations: es_dhb, effect size of difference in haemoglobin (intervention vs. control); s.e., standard error.
