# Supplementary material for: Health effects of micronutrient fortified dairy products and cereal food for children and adolescents: A systematic review
Source: PLoS One. 2019 Jan 23;14(1):e0210899. doi: 10.1371/journal.pone.0210899 (PMC6343890; doi:10.1371/journal.pone.0210899)
Supplement: S2 Fig — Studies with iron fortification included (n = 8 RCT with 11 pair-wise comparisons). Results are provided as risk ratio (RR, 95%-CI) of suffering from iron deficiency in the intervention group compared with the control group. Displayed subgroups: iron single MN fortification (1); iron dual MN fortification (2), iron multi MN fortification (3). (DOCX) [file pone.0210899.s002.docx]

**S2 Fig**. **Effect of iron-fortified dairy products and cereals on iron deficiency compared with non-fortified food.** Studies with iron fortification included (n=8 RCT with 11 pair-wise comparisons).

Results are provided as risk ratio (RR, 95%-CI) of suffering from iron deficiency in the intervention group compared with the control group. Displayed subgroups: iron single MN fortification (1); iron dual MN fortification (2), iron multi MN fortification (3).

**
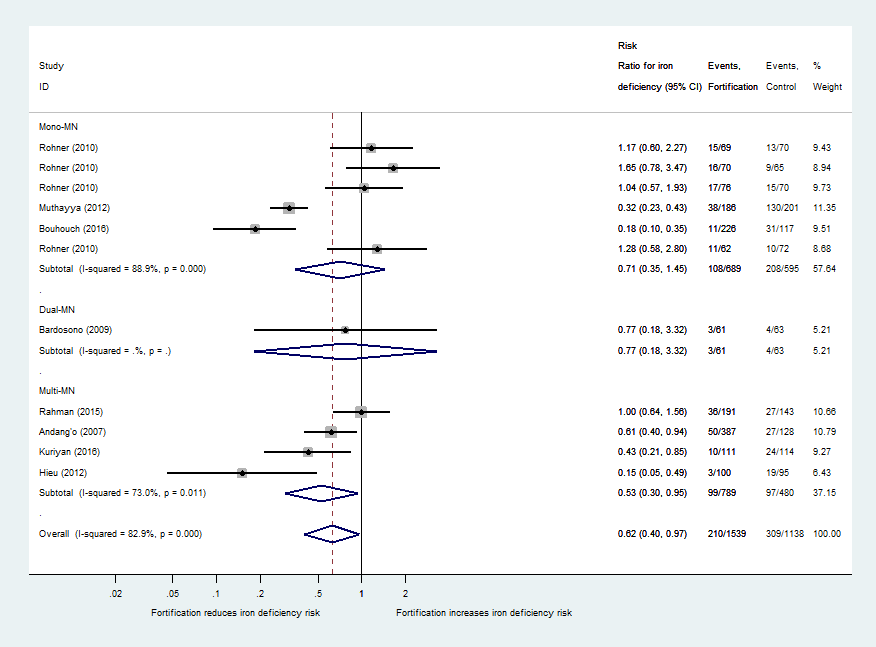
**
