# Supplementary material for: Health effects of micronutrient fortified dairy products and cereal food for children and adolescents: A systematic review
Source: PLoS One. 2019 Jan 23;14(1):e0210899. doi: 10.1371/journal.pone.0210899 (PMC6343890; doi:10.1371/journal.pone.0210899)
Supplement: S2 Table — (DOCX) [file pone.0210899.s004.docx]

**S2 Table**. **Inclusion and exclusion criteria of the systematic review**

|  | ***Inclusion criteria:*** |
| --- | --- |
| **Study design** | Randomised controlled trials |
| **Population** | Age: children and adolescents aged from 5 years up to 15 years;  all risk groups will be considered; any geographical location  any setting (e.g. meals at home; school feeding programs; community interventions) |
| **Intervention** | **Fortified milk products or cereals** (centrally fortified; may be in addition to other nutritional approaches, if these are applied in the intervention and control group, i.e. net nutritional difference between groups is fortified milk products or cereals):  Milk products include (beyond others) fresh milk; centrally processed milk; milk products (such as yoghurts, milk powder, cheese)  Cereals include:   - Fortified wheat flour, maize (corn), pearl millet grains, sorghum, oats, rye, buckwheat - Food preparations with fortified cereals include: porridge, gruel, “muesli”, bread, biscuits, sweet rolls, rusk   Micro-nutrients for fortification (for example: iron, Vitamin_X, zinc, iodine, folate, calcium, phosphorus, magnesium, selen, fluor,...) |
| **Control intervention** | Non-fortified food;  Non-fortified milk products or cereal products;  Other nutritional approaches, if these are applied in the intervention and control group (i.e. the net nutritional difference between groups is fortified milk products or cereals);  Co-interventions (e.g. deworming) accepted, if these are applied in the intervention and control group |
| **Outcome measures** | **Primary outcomes:** hemoglobin values (g/dl); anemia rates (i.e. blood parameters with direct health impact)  **Secondary outcomes:**  Blood parameters: iron stores (ferritin)  Health outcome:   - Growth; body weight; functional status; cognitive development; QOL - Morbidity (as measured with physical or mental health measures) - Mortality   Level of school performance; education; productivity;  Acceptability of fortified products;  Harms of fortified food; |

|  | ***Ex-clusion criteria:*** |
| --- | --- |
| **Study design** | Systematic reviews; case reports; observational studies (e.g. case-control-studies; cohort studies); “clinical” reviews (not SR); Guidelines; Abstracts; Editorials; commentaries. Letters; |
| **Population** | Excluded: children < 5yr of age; adolescents and adults > 15 years of age;  Excluded: children or adolescents with manifest disease (not due to MND, e.g. HIV, malaria), where fortified food may be part of the wider treatment regimen;  Excluded: Women in child bearing age, typically targeted via folate programs to reduce NTD of their babies |
| **Intervention** | Excluded: fortification of other staple food (e.g. salt; sugar, water, oil)  Excluded: fortified rice  Excluded: fortified condiments  Excluded: mere pulses and soy bean products; amaranth; (mixed products, e.g. “wheat plus soy”, are included)  Excluded: Nutritional interventions solely based on *supplementation*; “sprinkles” (as a form of home fortification); nutritional interventions solely based on *food based approaches;* bio-fortification  Excluded: “infant formula” (as defined in CODEX STAN 72-1986);  Excluded: fortification with components other than micro-nutrients (e.g. amino acids, fatty acids, enzymes)  Excluded: fortified supplementary food primarily aiming at augmentation of macro-nutrient density (e.g. ready to use therapeutic food, RUTF; fortified lipid based spreads)  Excluded: interventions to test different additives to study resorption / bioavailability of micronutrients (“before product” food technology studies) |
| **Control intervention** | specific nutritional approaches (e.g. energy dense spreads) or co-interventions that are only applied in the control group but not in the intervention group |
| **Outcome measures** | Excluded: Only surrogate parameters assessed (e.g. only MN blood levels; only bone mineral density); (exception: ferritin in combination with anemia/Hb) |
