# Supplementary material for: Health effects of micronutrient fortified dairy products and cereal food for children and adolescents: A systematic review
Source: PLoS One. 2019 Jan 23;14(1):e0210899. doi: 10.1371/journal.pone.0210899 (PMC6343890; doi:10.1371/journal.pone.0210899)
Supplement: S3 Table — Studies are listed alphabetically by author’s name. (DOCX) [file pone.0210899.s005.docx]

**S3 Table**. **Examples of excluded studies.** Studies are listed alphabetically by author’s name.

| **Study** | **Reason for exclusion** |
| --- | --- |
| Barbosa 2012 | This study evaluated the impact of the fortification of rolls with microencapsulated iron sulphate with sodium alginate on the haemoglobin levels in pre-schoolers as compared with controls. The double-blind randomised controlled trial comprised children aged 2-6 years with initial haemoglobin exceeding 9 g/dl from four not-for-profit day care centres randomly selected in the city of Sao Paulo - Brazil.  This study was excluded as the population inclusion criteria for age were not met. |
| Cabalda 2009 | The study sought to determine the efficacy of pandesal rolls baked from wheat flour fortified with iron, with or without vitamin A (VA), in improving anemic schoolchildren's iron and anthropometric status. Anemic 6-12-year-old Filipino children (n = 250) received two 60 g pandesal daily for 8 months. They were randomised into 1 of 4 groups: (1) iron-fortified (with hydrogen-reduced iron at 80 mg/kg, electrolytic iron at 80 mg/kg, or ferrous fumarate at 40 mg/kg), (2) iron and VA-fortified, (3) VA-fortified (at 490 RE/100 g), and (4) nonfortified flour. Haemoglobin (Hb) and zinc protoporphyrin (ZnPP) concentrations and weight and height were determined before and after intervention.  This study was excluded as no outcome data were provided which were suitable for data extraction to be included in a quantitative analysis. |
| Champak 2001 | The study objective was to determine the impact of single and combined interventions on nutritional status and scholastic and cognitive performance of schoolchildren. In a double-blind, randomised, placebo-controlled trial in 11 rural South African primary schools 579 children aged between 8 and 10 years were randomly allocated into six study groups, half of whom received anthelminthic treatment at the baseline. The outcome measures were anthropometric, micronutrient and parasite status, and scholastic and cognitive test scores.  This study was excluded as no reliable information about group sizes could be extracted. Thus, no conclusions could be drawn concerning treatment effects in the intervention group vs. control group. |
| Gonzalez-Rosendo 2010 | This study assessed a heme-iron concentrate product derived from swine haemoglobin to enrich the chocolate-flavoured filling of biscuits. The bioavailability of this source of heme-iron was assessed in adolescent girls from a rural area of Mexico. The placebo control (PC) group consisted of 35 teenagers with the highest baseline haemoglobin concentrations. The supplemented groups were randomised to receive biscuits fortified with iron sulphate (IS, n = 37) or heme-iron concentrate (HIC, n = 40).  This study was excluded for design reasons (randomisation was done as a bioavailability study between the two intervention groups; no randomised control group) |
| Huang 2009 | This study aimed to observe the different impacts of electrolytic iron, FeSO4, and NaFeEDTA on body iron store of anemic school students. Four hundreds anemic students at the age of 11-18 years were divided into four groups. Of these, three consumed different iron fortificants from wheat flour as food vehicle for six months and one consumed non-fortified flour (control).  This study was excluded for design reasons (no randomised controlled trial). |
| Zahrou 2016 | This study aimed to assess the impact of iodine deficiency (ID) on the intellectual development of Moroccan schoolchildren and to evaluate the effect of consumption of fortified milk on reducing ID. In a double-blind controlled trial conducted on schoolchildren, children were divided into two groups to receive fortified milk (30% of the cover of RDI iodine) or non-fortified milk for nine months.  This study was excluded for design reasons (no randomised controlled trial). |
