# Supplementary material for: Health effects of micronutrient fortified dairy products and cereal food for children and adolescents: A systematic review
Source: PLoS One. 2019 Jan 23;14(1):e0210899. doi: 10.1371/journal.pone.0210899 (PMC6343890; doi:10.1371/journal.pone.0210899)
Supplement: S4 Table — (DOCX) [file pone.0210899.s006.docx]

**S4 Table. Comparison of results of industry funded studies vs. non-industry funded studies.**

| **Outcome** | **All studies**  (24 RCT with 30 PWC^#^) | | **Industry funded studies***  (15 RCT with 19 PWC^#^) | | **Non-industry funded studies***  (9 RCT with 11 PWC^#^) | |
| --- | --- | --- | --- | --- | --- | --- |
| **Haemoglobin** | 14 RCT  (19 PWC) | **0.09 mg/dl** (95%-CI: -0.01 to 0.18) | 10 RCT  (13 PWC) | **0.08 mg/dl** (95%-CI: -0.04 to 0.19) | 4 RCT  (6 PWC) | **0.11 mg/dl** (95%-CI: -0.04 to 0.26) |
| **Anaemia** | 12 RCT  (17 PWC) | **OR 0.87** (95%-CI: 0.76 to 1.01) | 8 RCT  (11 PWC) | **OR 0.89** (95%-CI: 0.76 to 1.05) | 4 RCT  (6 PWC) | **OR 0.84** (95%-CI: 0.67 to 1.06) |
| **Iron deficiency** | 8 RCT  (11 PWC) | **OR 0.62** (95%-CI: 0.40 to 0.97) | 7 RCT  (10 PWC) | **OR 0.59** (95%-CI: 0.37 to 0.95) | 1 RCT  (1 PWC) | **OR 1.00** (95%-CI: 0.64 to 1.57) |
| **Iron deficiency anaemia** | 5 RCT  (5 PWC) | **OR 0.38** (95%-CI: 0.18 to 0.81) | 5 RCT  (5 PWC) | **OR 0.38** (95%-CI: 0.18 to 0.81) | No data | ---- |
| **Anthropometrics Stunting (height for age Z-score)** | 3 RCT  (4 PWC) | **0.02** (95%-CI: -0.07 to 0.11) | 2 RCT  (2 PWC) | **0.03** (95%-CI: -0.09 to 0.14) | 1 RCT  (2 PWC) | **0.01** (95%-CI: -0.14 to 0.16) |
| **Anthropometrics Wasting (weight for height Z-score)** | 1 RCT  (1 PWC) | **0.02** (95%-CI: -0.12 to 0.15) | No data | ---- | 1 RCT  (1 PWC) | **0.02** (95%-CI: -0.12 to 0.15) |
| **Cognitive and functional measures** | 8 RCT | **4** RCT: better results  **4** RCT: no improvement | 6 RCT | **2** RCT: better results  **4** RCT: no improvement | 2 RCT | **2** RCT: better results |
| **Physical measures** | 2 RCT | **2** RCT: no improvement | 2 RCT | **2** RCT: no improvement | No data | --- |
| **Morbidity** | 6 RCT | **3** RCT: improved health status  **3** RCT: no improvement | 3 RCT | **1** RCT: improved health status  **2** RCT: no improvement | 3 RCT | **2** RCT: improved health status  **1** RCT: no improvement |

* Industry funding comprises exclusive industry funding, mixed funding (e.g. industry together with public agencies) or no information was given. Non-industry funding includes: exclusive funding by public agencies or other funding sources (e.g. private foundations). ^#^PWC: pair-wise comparison within a randomised controlled trial (RCT)
