# Supplementary material for: Health effects of micronutrient fortified dairy products and cereal food for children and adolescents: A systematic review
Source: PLoS One. 2019 Jan 23;14(1):e0210899. doi: 10.1371/journal.pone.0210899 (PMC6343890; doi:10.1371/journal.pone.0210899)
Supplement: S5 Table — (PDF) [file pone.0210899.s007.pdf]

S5 Table. ROB assessment: applied criteria

2018_04_23 (modified acc. to CHB v5.2.0)

item	Type of Bias	Domain	High risk / low risk / unclear risk
Q1_rseq	Selection bias	sequence generation	0 (high): in-adequate method<br>1 (low): adequate method described<br>2 (unclear): incomplete information given
Q2_rconc	Selection bias	Allocation concealment	0 (high): in-adequate method<br>1 (low): adequate method described<br>2 (unclear): incomplete information given
Q3_blpp	Performance bias	Blinding of patients & personell	0 (high): in-adequate method<br>1 (low): adequate method described<br>2 (unclear): incomplete information given
Q4_blout	Detection bias	Blinding of outcome assessment	0 (high): in-adequate method<br>1 (low): adequate method described<br>2 (unclear): incomplete information given
Q5_compl	Attrition bias	Incomplete outcome data	0 (high): completeness not fulfilled: (< 80%) or imbalance between groups<br>1 (low): completeness fulfilled: >= 80% of participants analysed or missing values imputed and no imbalance between groups<br>2 (unclear): incomplete information given
Q6_report	Reporting bias	Selective outcome reporting<br>(i.e. systematic difference between reported and unreported outcomes)<br>(PO = primary outcome<br>KO: key outcomes<br>SP: study protocol)	0 (high): not all pre-specified PO reported;<br>OR one/more PO reported but measurements/analysis/subset of data not pre-specified;<br>OR reported PO not pre-specified<br>OR one/more outcomes reported incompletely;<br>OR report failed to include a key outcome expected (i.e. both outcomes are key):<br>• Serum markers (if iron fortification: haemoglobin; if no iron fortification: other MN-status, e.g. VitD-status, bone mineral density, is suitable)<br>• Height/weight/z-scores OR functional measures (incl. cognitive development) OR morbidity/mortality<br>1 (low): study protocol available; OR report contains all expected outcomes (incl. pre-specified; may be uncommon)<br>2 (unclear): incomplete information given
