# Supplementary material for: Health effects of micronutrient fortified dairy products and cereal food for children and adolescents: A systematic review
Source: PLoS One. 2019 Jan 23;14(1):e0210899. doi: 10.1371/journal.pone.0210899 (PMC6343890; doi:10.1371/journal.pone.0210899)
Supplement: S1 SP — (PDF) [file pone.0210899.s008.pdf]

PROSPERO International prospective register of systematic reviews

Health effects of micronutrient fortified milk and cereal food for children and adolescents: a systematic review (study protocol)

Klaus Eichler, Hess Sascha, Claudia Twerenbold, Magalie Sabatier, Flurina Meier, Simon Wieser

Citation

Klaus Eichler, Hess Sascha, Claudia Twerenbold, Magalie Sabatier, Flurina Meier, Simon Wieser. Health effects of micronutrient fortified milk and cereal food for children and adolescents: a systematic review (study protocol). PROSPERO 2016:CRD42016039554 Available from
http://www.crd.york.ac.uk/PROSPERO_REBRANDING/display_record.asp?ID=CRD42016039554

Review question(s)

Which health effects are induced by fortified milk products and cereals in micronu-trient deficient children and adolescents from 5 to 15 years of age compared to non-fortified milk products and cereals?

Is there any difference in health effects of fortified milk products vs. fortified cereal food?

Is there any difference in health effects of mono- vs. dual/multi-micronutrient fortified strategies?

Searches

We will search in the following electronic databases: MEDLINE (OVID Interface), EMBASE, the Cochrane Library.

In addition, we will screen homepages of organisations engaged in nutrition projects in developing countries (e.g. WHO, United Nations [World Food Programme, Unicef, Milleni-um Development Goals], The World Bank, Pakistan National Nutrition Survey; Interna-tional Clinical Epidemiology Network [www.inclen.org]; Global Alliance for Improved Nutri-tion, GAIN, [www.gainhealth.org]); Micronutrient Initiative (www.micronutrient.org); Bill & Melinda Gates Foundation (www.gatesfoundation.org).

Furthermore, we will undertake Internet searches using Google Scholar, will scan the ref-erence lists of included papers and will perform citation searches. Experts in the field will also be contacted for information on potential studies. Additional information and research can be provided by manufacturers of fortified food (e.g. Nestlé).

We will primarily use search terms that have been applied in more recent research projects for fortified food issues (for example: “milk”[MeSH]; “edible grain”[MeSH]; cereal*[Title/Abstract]; fortif*; child*; “adolescent”[MeSH]; micronutrients[MeSH])

Applied search terms will be tested in a pilot search.

Search terms will then be refined in a stepwise approach in close collaboration with a Medical Librarian with expertise in systematic review searching.

The draft search strategy for MEDLINE is available in the published protocol.

Language of publication: No language restriction

Years of publication: To ensure that the retrieved literature is relevant to current fortification practice we restrict the searches to more current literature (from 1980 onwards).

Types of study to be included

Inclusion: randomised controlled trials (RCT)

Exclusion:

Systematic reviews; case reports; observational studies (e.g. case-control-studies; cohort studies); “clinical” reviews (not SR); Guidelines; Abstracts; Editorials; Commentaries; Letters (exception: publication of an RCT as “letters to the Editor”);

Existing systematic reviews, relevant for the planned review, may also be used to find ad-ditional primary studies.

Condition or domain being studied

Micronutrient (MN) deficiencies imply a considerable burden of disease for many middle and low income countries, resulting for example in reduced growth, high anemia prevalence or increased infection rates.

Participants/ population

Inclusion: Age: children (age 5 to 11.9 years) and adolescents (age 12 to 15 years); girls and boys; all risk groups will be considered; studies with mixed population groups are only included, if data for age group 5 to15 years are reported or if information is available, that more than half of the participants lie within the age range of 5 to 15 years. Sensitivity analyses will be carried out if marginal decisions are made.

Exclusion: Children < 60 months of age; adolescents and adults > 15 years of age; children or adolescents with manifest disease, where fortified food may be part of the wider treatment regimen.

Intervention(s), exposure(s)

Inclusion: Fortified milk products and fortified cereals, each centrally processed

Milk products include:

• fresh milk; centrally processed milk; milk products (such as yoghurts, milk powder, cheese)

Cereals include:

• Fortified wheat flour, maize (corn), pearl millet grains, sorghum, oats, rye, buck-wheat

• Food preparations with fortified cereals include: porridge, gruel, “muesli”, bread, biscuits, sweet rolls, rusk

Micronutrients for fortification include:

• iron, vitamins, zinc, iodine, calcium, folate, phosphorus, magnesium, selen, fluor

Fortification strategies include:

• Different fortification strategies (i.e. mono-, dual, multi-micronutrient fortification) with different micronutrients

Exclusion:

• Nutritional interventions solely based on supplementation; point-of-care fortification (e.g. via “sprinkles” as a form of home fortification); bio-fortification; nutritional in-terventions solely based on food based approaches;

• Rice, as fortification of rice with several MN is addressed by an ongoing Cochrane review

• Pulses (seeds of leguminous plants; CODEX STAN 171-1989) and soy protein products (e.g. tofu; soya milk; CODEX STAN 175-1989); amaranth;

• “infant formula” (CODEX STAN 72-1986);

• Fortification with components other than micro-nutrients (e.g. amino acids, fatty acids, enzymes)

• Fortification of other staple food (e.g. salt; sugar, water, oil)

• Fortified condiments

• Fortified supplementary food primarily aiming at augmentation of macro-nutrient density (e.g. ready to use therapeutic food, RUTF; fortified lipid based spreads)

• Interventions to test different additives to study resorption / bioavailability of micro-nutrients (“before product” food technology studies)

Comparator(s)/ control

Inclusion: non-fortified food; non-fortified milk or cereal products; or other nutritional approaches, if these are applied in the intervention and control group (i.e. the net nutritional difference between groups is fortified milk products or cereals)

Exclusion: studies with specific nutritional approaches (e.g. energy dense spreads) that are only applied in the control group but not in the intervention group

Context

Inclusion: any study setting (e.g. meals at home; school feeding programs; community interventions)

Outcome(s)

Primary outcomes

Blood parameters with direct health impact: hemoglobin values (g/dl); anemia rates, with anemia thresholds as defined in the primary study

Secondary outcomes

Blood parameters: iron stores (ferritin)

Health outcome:

- Growth; body weight; functional status; cognitive development; quality of life (QOL)

- Morbidity (as measured with physical or mental health measures)

- Mortality (at end of study)

Level of school performance; education; productivity (at longer follow-up)

Acceptability of fortified products;

Harms of fortified food;

Data extraction, (selection and coding)

Title and abstract screening:

Three trained review author pairs will screen titles and abstracts for relevance. Within each pair, screening will be done independently by each reviewer. Disagreements will be resolved by consensus. Unclear cases will be discussed with a senior reviewer.

Full text assessment:

Potentially relevant studies will be ordered and the full text copy will be assessed for final decision by one reviewer, with decisions checked independently by a second reviewer.

If data of a specific population were published in several papers or if follow-up data were presented, we will include each population only once.

Data extraction:

Using predefined Excel databases data will be extracted by one reviewer and checked independently by a second

reviewer. To ensure consistency between reviewers, prior training sessions will be held. Discrepancies will be resolved by discussion.

Data to be extracted:

Study details:

• study identifier, author, year, aim of the study, study design, location, setting (e.g. level of population recruitment), length and completeness of follow up, kind of sponsorship (e.g. public, industry, none)

Participant details:

• number of participants in each group, age, sex, in-/exclusion criteria of the primary study,

Features of intervention and control:

• daily amount of fortified MN, determined as daily difference between intervention and control group; composition of MN; fortification strategy; comparator food;

Study results (primary outcome; for intervention group and control group):

• Hb change mean (g/dl), SD

• number of persons with anemia, at end of follow-up;

Study results (secondary outcome; for intervention group and control group):

• Ferritin end of follow-up mean/median (micro-g/l), SD

• number of persons with iron deficiency, at end of follow-up;

• Growth (mean change, SD)

• body weight (mean change, SD)

• anthropometric measures (weight or height gain; Z-scores: weight-for-height, height-for-age)

• functional status (as measured with physical health measures)

• cognitive development (as measured with mental health measures)

• motor skill development (as defined in the primary studies)

• quality of life, QOL (as measured with validated QOL measures, such as SF-36, EQ-5D)

• Morbidity (rates of reported diseases at end of study follow-up: e.g. diarrhoea, respiratory infections, referral to hospital)

• Mortality rates at end of study follow-up

• Level of school performance; education; productivity (at longer follow-up)

• Acceptability of fortified products;

• Side-effects of fortified food (e.g. nausea, diarrhoea)

Data may also be extracted on other items, which will be deemed as important after closer inspection of studies that

meet the inclusion criteria.

Risk of bias (quality) assessment

Risk of bias (ROB) assessment forms will be developed on Microsoft Excel using current guidelines.

One reviewer will assess risk of bias in individual studies with a component approach ex-ploring methodological quality on the study level (adequate generation of random se-quence, concealment of allocation, blinding) as well as on the outcome level (incomplete outcome data due to attrition; selective outcome reporting).

Risk of bias assessment will be carried out by one reviewer and checked independently by a second reviewer. Disagreements will be resolved by consensus. Unclear cases will be discussed with a third reviewer. Reviewers will not be blinded to studies.

We will apply the following definitions for ROB assessment:

Criterion 1: Randomisation: Adequate sequence generation?

• Low risk of bias: description of an adequate method for randomised studies

• high risk of bias: description of in-adequate method

• unclear risk of bias: incomplete information given

Criterion 2: Randomisation: Allocation concealment?

• Low risk of bias: description of an adequate method for randomised studies

• high risk of bias: description of in-adequate method

• unclear risk of bias: incomplete information given

Criterion 3: Blinding?

• Low risk of bias: blinding described for randomised studies

• high risk of bias: description of in-adequate blinding method

• unclear risk of bias: incomplete information given

Criterion 4: Incomplete outcome data addressed? (attrition)

• Low risk of bias: If completeness fulfilled: (>= 80% of participants analysed) or missing values imputed.

• high risk of bias: If completeness not fulfilled: (< 80% of participants analysed)

• unclear risk of bias: incomplete information given

Criterion 5: Are typical outcomes or data reported? (i.e. no selective outcome reporting)

Definition of basic outcome domains to be reported for Criterion 5:

1) MN-status; serum markers (if iron fortification: haemoglobin, anemia rates, or Ferritin; if no iron fortification: other MN-status, e.g. VitD-status, is suitable)

2) Height/weight/z-scores; functional measures (incl. cognitive development)

3) morbidities / mortality

• Low risk of bias: at least 2 of 3 typical outcomes have to be reported (i.e. MN-status and 2; or MN-status and 3; or 2 and 3)

• high risk of bias: less than 2 of 3 typical outcomes are reported

• unclear risk of bias: incomplete information given

Risk of bias assessment will be presented in a transparent table format to allow the reader full insight into methodologic strengths and shortcomings of included studies. Thus, risk of bias assessment will be used for descriptive purposes to provide an evaluation of the overall methodological quality of the included studies. In addition, it can be used for pre-specified subgroup analyses.

Strategy for data synthesis

Narrative analysis:

A systematic and narrative analysis of the included studies will be presented in the text and in a tabulated form. This will allow for a systematic overview about study characteristics (e.g. design, study aim) and features of the included population, setting, kind of inter-vention, and outcome measures to judge similarities and differences between studies. This presentation format will also give an overview over the effectiveness of interventions to improve MN deficiencies by fortified milk products and cereals.

Statistical meta-analysis:

If no relevant heterogeneity in terms of populations, interventions, comparators and outcomes between studies exist, an analysis with statistical pooling will be performed.

Conditions to be present for statistical pooling:

• Design: We do not expect heterogeneity (only RCT included)

• Population: We deem the included age group of 5 to 15 years as sufficiently homogenous for pooling

• Intervention: Studies with mono- and dual/multi-nutrient approaches will be pooled (but this feature will be included in the pre-specified subgroup analysis)

• Comparator: no restriction for pooling, as long as the net difference between groups is MN-fortification

• Outcome: no restriction for pooling of blood values; for functional outcome pooling of the standardised mean difference, depending on the data available

• Risk of bias: low risk and high risk of bias studies will be pooled (but this feature will be included in the pre-specified subgroup analysis)

For pooling of continuous variables we will compute weighted mean differences (WMD) and 95%-confidence intervals (CI) with the inverse variance method. For example, for analysis of the primary outcome hemoglobin change we will use the mean change in the intervention and in the control group and their pooled standard deviation (SD). For func-tional outcome we will calculate the standardised mean difference, if different measure-ment scales have been used in the primary studies.

For pooling of binary data, we will calculate risk ratios and 95%-CI.

Heterogeneity between trials will be calculated with I2, that is the percentage of the total variation in estimated effects that is due to heterogeneity rather than chance 27 (0%-40% might not be important; 30% to 60% may represent moderate heterogeneity; 50% to 90% may represent substantial heterogeneity; 75% to 100%: considerable heterogeneity). As we expect at least moderate statistical heterogeneity between trials, we will apply a random effects model.

If the sample size decreased during the study, we will use the lower sample size at the end of the study. If mean hemoglobin change per group and SD is not reported, we will calculate change as the difference of baseline and final values for intervention and control group and apply the SD of final values.

If only 95%-CI of mean values are reported, we will convert them to SD assuming normal distribution. To check results for robustness, we will also calculate WMD for final hemoglobin values of both randomised study groups at the end of follow-up. If authors report only medians for continuous data (e.g. for ferritin levels), we will not include those data in a meta-analysis, but report distribution of median values and inter-quartile-ranges (IQR).

Analysis of subgroups or subsets

Subgroup analysis:

Furthermore, analysis of pre-specified subgroups to explore the influence of possible modifying factors on the outcome will be performed. Pre-specified subgroups include:

• fortified milk vs. fortified cereal food;

• single- vs. dual/multi-micronutrient fortification strategy

• studies from high vs. studies from low/middle-income countries;

• studies with low risk of bias vs. studies with intermediate/high risk of bias

Meta-regression analysis:

If enough data are available, we will perform a meta-regression analysis weighted for the inverse of the variance of the outcome to further explain possible heterogeneity. With this approach we will evaluate the unique contribution of other a priori chosen independent factors on the primary outcome (dependent variable). Pre-specified factors for meta-regression include:

• mean haemoglobin level at start of study

• daily amount of consumed MN from fortified food;

• length of study follow-up;

• completeness of study follow-up

Assessment of meta-bias:

Depending on the number of included primary studies, an assessment of meta-bias via a graphical method (funnel plot) may be performed. This can give an indication if a possible publication bias may have influenced overall review results.

Statistical analyses will be performed using the STATA SE 14 software package (Stata-Corp. 2007. Stata Statistical Software, College Station,Texas, USA).

Confidence in cumulative estimate:

To make an overall rating of confidence in effect estimates across single outcomes, we will apply the GRADE's approach to rating confidence in estimates of effects.

This will be done for the primary outcome (haemoglobin change; anemia rates), as well as for relevant secondary outcomes (growth; body weight; functional status; cognitive development; morbidity).

Dissemination plans

Submission of a manuscript to a scientific journal.

Contact details for further information
Professor Eichler

ZHAW Zurich University of Applied Sciences

Winterthur Institut of Health Economics

Health Services Research

Gertrudstrasse 15

Postfach 958

CH-8401 Winterthur

Schweiz

eich@zhaw.ch

Organisational affiliation of the review
Winterthur Institute of Health Economics, Zurich University of Applied Sciences, Ger-trudstrasse 15, CH-8401 Winterthur, Switzerland

https://www.zhaw.ch/de/sml/institute-zentren/wig/

Review team
Professor Klaus Eichler, Winterthur Institute of Health Economics
Mrs Hess Sascha, Winterthur Institute of Health Economics
Dr Claudia Twerenbold, Winterthur Institute of Health Economics
Mrs Magalie Sabatier, Nestlé Research Center
Mrs Flurina Meier, Winterthur Institute of Health Economics
Professor Simon Wieser, Winterthur Institute of Health Economics

Collaborators
Dr Martina Gosteli, Medical Librarian, Careum, University of Zurich, Switzerland

Anticipated or actual start date
02 May 2016

Anticipated completion date
28 February 2017

Funding sources/sponsors
The study is financially supported by the Nestlé Research Center, Lausanne, Switzerland. M. Sabatier is an employee of the Nestlé Research Center. The funding body commented on the final draft of the review protocol but did not make final decisions regarding the design of the review, the planned data collection and the analysis plan.

Conflicts of interest
The study is financially supported by the Nestlé Research Center, Lausanne, Switzerland. M. Sabatier is an employee of the Nestlé Research Center. The funding body commented on the final draft of the review protocol but did not make final decisions regarding the design of the review, the planned data collection and the analysis plan.

Language
English

Country

Switzerland

Subject index terms status
Subject indexing assigned by CRD

Subject index terms
Adolescent; Animals; Child; Dietary Supplements; Edible Grain; Humans; Micronutrients; Milk; Trace Elements

Stage of review
Ongoing

Date of registration in PROSPERO
26 May 2016

Date of publication of this revision
09 May 2017

Stage of review at time of this submission	Started	Completed
Preliminary searches	Yes	Yes
Piloting of the study selection process	Yes	Yes
Formal screening of search results against eligibility criteria	Yes	Yes
Data extraction	Yes	Yes
Risk of bias (quality) assessment	Yes	Yes
Data analysis	Yes	Yes

PROSPERO
International prospective register of systematic reviews
The information in this record has been provided by the named contact for this review. CRD has accepted this information in good faith and registered the review in PROSPERO. CRD bears no responsibility or liability for the content of this registration record, any associated files or external websites.
